# Overview of Crosstalk Between Multiple Factor of Transcytosis in Blood Brain Barrier

**DOI:** 10.3389/fnins.2019.01436

**Published:** 2020-01-21

**Authors:** Marco Tjakra, Yeqi Wang, Vicki Vania, Zhengjun Hou, Colm Durkan, Nan Wang, Guixue Wang

**Affiliations:** ^1^Key Laboratory for Biorheological Science and Technology, Ministry of Education, State and Local Joint Engineering Laboratory for Vascular Implants, Bioengineering College of Chongqing University, Chongqing, China; ^2^The Nanoscience Centre, University of Cambridge, Cambridge, United Kingdom

**Keywords:** blood brain barrier, transcytosis, developmental, mechanical stress, cytokines, miRNA, physicochemical, tight junctions

## Abstract

Blood brain barrier (BBB) conserves unique regulatory system to maintain barrier tightness while allowing adequate transport between neurovascular units. This mechanism possess a challenge for drug delivery, while abnormality may result in pathogenesis. Communication between vascular and neural system is mediated through paracellular and transcellular (transcytosis) pathway. Transcytosis itself showed dependency with various components, focusing on caveolae-mediated. Among several factors, intense communication between endothelial cells, pericytes, and astrocytes is the key for a normal development. Regulatory signaling pathway such as VEGF, Notch, S1P, PDGFβ, Ang/Tie, and TGF-β showed interaction with the transcytosis steps. Recent discoveries showed exploration of various factors which has been proven to interact with one of the process of transcytosis, either endocytosis, endosomal rearrangement, or exocytosis. As well as providing a hypothetical regulatory pathway between each factors, specifically miRNA, mechanical stress, various cytokines, physicochemical, basement membrane and junctions remodeling, and crosstalk between developmental regulatory pathways. Finally, various hypotheses and probable crosstalk between each factors will be expressed, to point out relevant research application (Drug therapy design and BBB-on-a-chip) and unexplored terrain.

## Introduction

### Blood Brain Barrier Concept and Constituents

The neurovascular unit is a complex system of blood vessels and nerves, together with neighboring cells and the extracellular matrix. There are numerous similarities, functions, interactions, and remodeling process interconnected between these two systems (vascular and nervous) in the body. A prominent example is the formation of the blood brain barrier (commonly abbreviated as BBB). The BBB is a complex mixture of various transport systems located between blood vessels and the brain. It is predominantly composed of endothelial cells, neurons, oligodendrocytes, pericytes, astrocytes, microglia, and surrounded by the extracellular matrix which is mainly composed of collagen and laminin. The primary function of the BBB is to provide a safety mechanism to prevent potentially harmful material from entering the brain, while still enabling the transcytosis of nutrients and signaling factors. Failure to maintain the BBB integrity results in abnormalities, mainly infectious diseases such as meningitis, multiple sclerosis, neurodegenerative diseases, and a plethora of brain disorders. Understanding the exact mechanisms of transcytosis in the BBB will provide useful insights for diseases and their possible treatments.

Among this complex mixture of cells, endothelial cells (ECs) are squamous cells that form the lining wall of the vascular system. The differentiation of endothelial cells is organ and tissue specific, modified via responses of the appropriate gene expression toward hemodynamic forces, extracellular stress, interaction with adjunct cells, and matrix secretions (Marcu et al., 2018). Brain microvasculature endothelial cells (BMECs) have an adaptation to form tight barriers and also active transport protein, such as P-glycoprotein (Stebbins et al., 2015). Expression of p-glycoprotein at BMECs, which is one of the efflux transporters, shows dependency to β-catenin upregulation (Lim et al., 2008). This clearance process showed that transcytosis in the BBB can be accomplished from abluminal to luminal sides. The response and behavior of BMECs also shows dependence on cytokine stimuli (Carroll et al., 2015). The primary formation of BBB tight junction is also regulated by ECs, together with other cells. Pericyte is interconnected with ECs in microvasculature, and act as the co-regulator of ECs. In the BBB, this type of cell plays an important role as one of the supporting cell types for BBB integrity and permeability via cell to cell communication and ECM to cell communication. Pericytes have been shown to regulate the differentiation of ECs into HBMECs. These cells also interact with astrocytes to induce polarization of astrocytes surrounding the blood vessel. A lack of pericytes in the mouse model and cell culture experiment causes increased BBB permeability. Treatment of imatinib, which depletes pericytes, inhibits the release of tracers from ECs to the brain via transcytosis. Thus it can be inferred that pericytes have a role in regulating transcytosis in the BBB (Armulik et al., 2010). Astrocytes are part of the modified glial cells. The primary role of these cells is to support neurons. In the BBB complex, the foot of astrocytes encircles ECs and blood vessels. Astrocytes also enable nutrients transport and delivery to neurons. A recent study showed astrocytes may promote blood flow and microvasculature remodeling (Figley and Stroman, 2011). One of the prominent mechanisms for tight junction regulation by astrocytes is by regulating the accumulation of agrin, a heparin sulfate proteoglycan which is important for BBB integrity in the basal lamina (Abbott et al., 2006).

**FIGURE 1:**
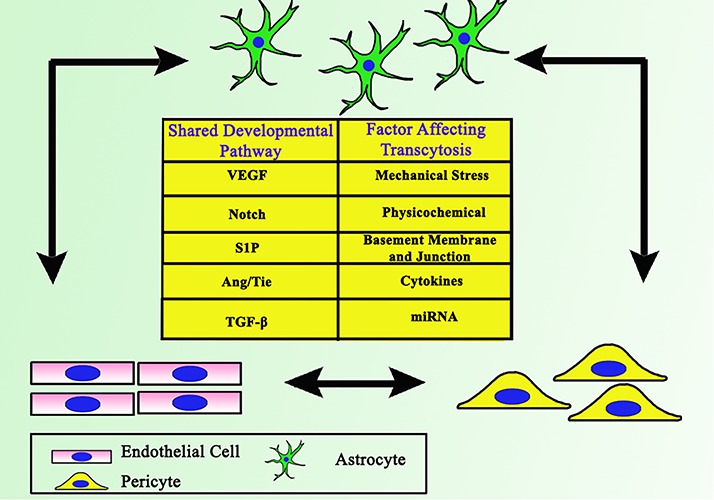
Close interaction between ECs, astrocytes, and pericytes as the backbone of BBB. Together they share some common developmental pathways, which involves each cell to support one another.

However, the exact mechanism whereby transcytosis may be affected by co-regulation between ECs, pericytes, and astrocytes is still unclear (Figure 1). Various relevant factors also still need further elucidation. This review will focus on revealing and summarizing current findings of interaction between ECs, pericytes, and astrocytes as well as the crosstalk of factors which may affect transcytosis in the BBB.

### Paracellular and Transcellular BBB Permeability

Molecular transport across the BBB is highly dependent on its permeability, which is defined by the paracellular pathway (molecules cross through ECs junctions) and the transcellular pathway (endocytosis followed by endosomal rearrangement and exocytosis from the cell). These mechanism contributes to the brain’s nutrition supply, as comprehensively reviewed by Lalatsa and Butt (2018). BMECs have a specialized tight junction in order to prevent undesirable paracellular transport and consequently direct the necessary molecules by a specific transcellular pathway (Zhou et al., 2019). The importance of transcytosis is emphasized under certain conditions including hypertension and strokes (Knowland et al., 2014). When pathogenesis occurs, the primary response is upregulation of caveolin, which facilitates caveolae assembly (Knowland et al., 2014). A similar finding in the case of multiple sclerosis also stresses the significant upregulation of caveolae-mediated transcytosis (Lengfeld et al., 2017). This response may be a reaction to facilitate the recovery process, which requires a higher supply of nutrients, as well as clearance of toxic materials.

**FIGURE 2:**
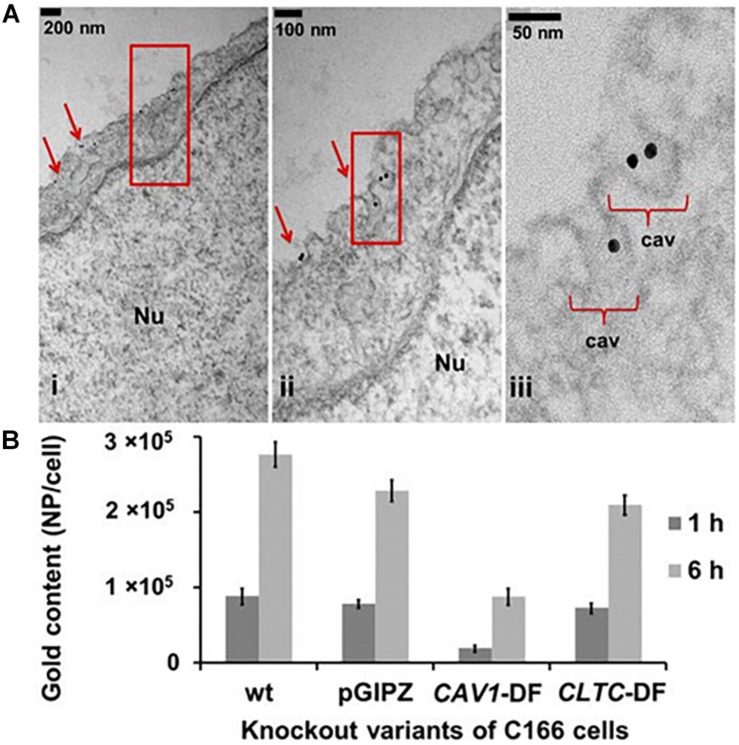
**(A)** Caveolae formation in endothelial cells. Nu, nucleus. **(B)** Caveolae is the main endocytosis pathway of ECs compared to clathrin. Uptake of spherical nucleic acids (SNAs) as the nanocarrier showed 60% reduction in the Cav-1 deficient cells, also showed 10% reduction in clathrin heavy chain (CLTC) deficient cells. Reproduced with permission and courtesy of Chad Mirkin (Choi et al., 2013).

Transcellular pathways in cells are categorized into clathrin mediated and non-clathrin mediated. Detail mechanism in several types of transcytosis can be studied extensively in the comprehensive review by Pulgar (2019) and Villaseñor et al. (2019). Non-clathrin mediated transport makes use of dynamin, coat proteins, small GTPases, and RhoGAP proteins. Caveolae-mediated transcytosis is one of the non-clathrin mediated pathway. Transportation between cells using extracellular vesicles (EVs) is very important during development and maintenance of the BBB. A recent study showed atheroprotective intercellular communication via EVs between ECs and smooth muscle cells through miRNAs regulation (Hergenreider et al., 2012). Based on these interactions, the transport pathway plays an essential role in intercellular communication. Which brings us to question, is there any interaction between paracellular and transcellular pathways? In the case of water transport, lack of protein transporter in the paracellular path may significantly impair the transcellular path (Kawedia et al., 2007). It has been elucidated that Cav-1 also plays a part in the regulation of TJ protein expression in HBMECs (Song et al., 2007), indicating a central role for the transcellular pathway in the BBB maintenance (Figure 2).

### Caveolae Biosynthesis

There are two main proteins which play a major role in caveolae biosynthesis. The first is caveolin, along with multiple isomers (Cav-1, Cav-2, and Cav-3). The second one is cavin, which currently has four known isomers. These two components are an essential part of caveolae, and the absence of either one will significantly suppress the amount and function of caveolae. Lipodystrophic phenotype has been observed both in mice and humans lacking caveolae, suggesting its importance for lipid transcytosis. A recent discovery has showed that binding of these components with phosphatidylserine plays a crucial role in caveolae formation (Hirama et al., 2017). Cav-1 and Cav-2 will form 8S oligomer on endoplasmic reticulum, followed by transport via COP II (coat protein complex II) to the Golgi. In this place, 8S Cav-1/Cav-2 will undergo oligomerization with cholesterol forming the 70S subunit, followed by transport to plasma membrane (Hayer et al., 2010).

In the context of the BBB, HBMECs have been modified to limit the number of caveolae, thus limiting leakiness and transcytosis. One reason for this regulation is the role of Mfsd2a, which is a facilitator at the cell membrane to transport the LPC-DHA supply to the brain. In contrast with the increasing lipid content, the cell will undergo suppression of caveolae amounts (Andreone et al., 2017). Meanwhile, elevated caveolae occurrence is a sign of pathogenesis (Table 1), indicating BBB leakiness with interaction of several other factors of transcytosis (Gu et al., 2012). However, attenuation of caveolae expression inhibits the expression of TJ proteins (Song et al., 2007) and also accelerates neurodegeneration and aging (Head et al., 2010). Keeping the balance of caveolae number for appropriate amount of transcytosis has been a challenging aspect for maintaining BBB stability.

**TABLE 1:** Several diseases with abnormality concerning transcytosis, as well as abnormality of the Cav-1 as the component of endocytosis.

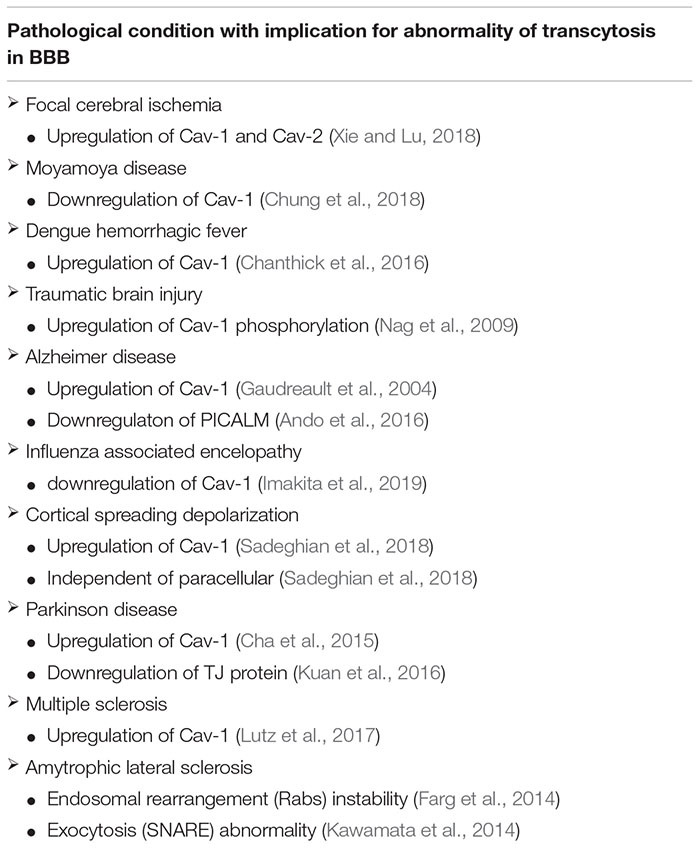

## Shared Developmental Pathway

### Vascular Endothelial Growth Factor (VEGF)

Vascular endothelial growth factor (VEGF) has cytoprotective effects on ECs by preventing apoptosis, mediated through phosphatidyl inositol 3-kinase (PI3K)/Akt pathway (Ferrara et al., 2003). Action of VEGF in angiogenesis is prominent, but for maintenance at the latter stages of development, pericytes will take over this function. Instead of maintaining, VEGF reduces barrier robustness through the nitric oxide synthase (NOS)/cGMP-dependent pathway (Mayhan, 1999). The activation of eNOS in Human Umbilical Vein Endothelial Cells (HUVECs) is also crucial for caveolae formation (Bai et al., 2017). Endocytosis of VEGF receptors are also caveolae dependent, inferred from a study of leukemia cell line (Caliceti et al., 2014). *In vivo* retina study using *Macaca fascicularis* shows administration of VEGF will induce angiogenic phenotype both in ECs and pericytes, thus it might be resulting in BBB instability in adults (Witmer et al., 2004). This side effect can be neutralized by administration of Ang1, which attenuates the activity of MMP-2 and MMP-9, without disturbing the angiogenesis in mice cerebrovascular (Valable et al., 2005). Activation of VEGF/PI3K/Akt pathway may induce actin reorganization in human angioma cells (Wang et al., 2011), a process known to be crucial for endocytosis and endosomal rearrangement (Podar and Anderson, 2008; Römer et al., 2010; Coelho-Santos et al., 2016). This might be one of the ways for VEGF controlling caveolae and transcytosis in the ECs. In the early symptoms of stroke and cerebral ischemia, regions of the brain can end up in hypoxic conditions. During hypoxia, VEGF will be secreted from the pericytes which affects claudin-5 and BBB integrity via paracellular pathway (Bai et al., 2015). Other secreted cytokines such as IL-6 and G-CSF attenuates BBB transcellular robustness via an unknown mechanism. Another study also highlights astrocytes role in BBB integrity attenuation for VEGF-A secretion during pathological condition (Argaw et al., 2012). Balance between VEGF activities to properly upregulate transcytosis while maintaining BBB stability still needs further investigation.

### Platelet-Derived Growth Factor (PDGF)-B/PDGF Receptor Beta (PDGFRβ)

At early stages of vessel formation, tip ECs will secrete PDGF-B to promote the recruitment of pericyte progenitor cells. This mitogen growth factor will be detected by PDGFRβ on the pericytes, leading the migration to tip ECs in the process of angiogenesis (Hellström et al., 1999). The expression will gradually decrease following vessel maturation, but irregularities will arise in the pathological conditions of several diseases as indicated by the increasing PDGF-B expression in mature vasculature (Gallini et al., 2016). This pathway still persists in the postnatal angiogenesis, indicating an important communication between pericytes and endothelial progenitor cells (EPCs) (Baumgartner et al., 2010). Lack of pericytes caused by diminished signaling of PDGF-B/PDGFRβ also showed fatality in mice phenotypes (Lindahl et al., 1997). In the neurovascular unit within adult mice, the expression of PDGFRβ exclusively persists only at pericytes (Winkler et al., 2010), differing from humans which also retain it in general ECs (Muhl et al., 2017). Transcription factor Foxf2 maintains PDGFRβ expression specifically in brain pericytes to support BBB integrity (Reyahi et al., 2015), indicating the role of the FOX family for maintaining the BBB. Endocytosis receptor Ephrin-B2 supports the internalization and also signaling of PDGFRβ in mice vascular smooth muscle cells (Nakayama et al., 2013), leaving room for further study in brain pericytes. Reactivation of PDGF-B/PDGFRβ signaling through administration of TGF-β can restore the function of the BBB after focal cerebral ischemia (Shen et al., 2018), indicating a crosstalk shared by these two pathway. *In vitro* experiment also showed protective effects of PDGF-BB on astrocytes through activation of antioxidant mechanism (Cabezas et al., 2018). Mice model also support this findings, emphasizing astrocytes roles to recover neuronal damage after hemorrhage (Zhou et al., 2019). Another complementary communication is the PDGF-D/PDGFRβ signaling which is supported by the co-receptor Neuropilin1 (NRP1) in ECs (Muhl et al., 2017). This communication involves NRP1 translocation, indicating a regulation for other pathways involving NRP1. NRP1 is also a co-receptor for the VEGF signaling pathway, indicating a crosstalk between these two pathways. NRP1 also regulates HMGB1, which induces caveolae formation in general ECs (Ma et al., 2019). Possibly PDGF signaling is able to manage transcytosis via this pathway, additionally activating a regular PI3K/AKT pathway for actin dynamic regulation.

### Transforming Growth Factor-β (TGF-β)

Transforming growth factor (TGF-β) plays an important role in angiogenesis together with VEGF. These cytokines have a range of different effects on ECs depending on the conditions: TGF-β may induce apoptosis via MAPK pathway on general ECs, while VEGF will protect general ECs from apoptosis (Ferrari et al., 2009). The process of apoptosis may induce vascular remodeling, which includes vessel pruning and maturation. Thus the role of TGF-β is indispensable within normal vessels. TGF-β1 dimer starts by binding with TGF-β receptor II, followed by TGF-β receptor I. This heterotetramer complex undergoes phosphorylation, subsequently activating Smad transcription factors: Smad2/3 will be activated first, forming a heterocomplex with Co-Smad Smad4. Subsequent transport of this complex to the cell nucleus may regulate expression of target genes (Daly et al., 2008). Transport of TGF-β receptor in HeLa cells model is known to be dependent on clathrin and caveolae, including the novel endosomal fusion between two vesicles which are regulated by Rab5 (He et al., 2015). Expression of TGF-β maintains cerebrovascular integrity by regulating N-cadherin expression in cooperation with Notch signaling (Li et al., 2011). However, activation of TGF-β also upregulates α-SMA (Smooth Muscle Actin) and actin in the brain pericytes, as well as the VEGF, MMP-3, and MMP-9 which promotes barrier instability (Thanabalasundaram et al., 2011). This is an issue requiring further investigation. TGF-β expression in brain pericytes has showed upregulation via Foxf2 expression (Reyahi et al., 2015). Treatment of brain pericytes with bFGF (basic Fibroblast Growth Factor) may promote expression of desmin, vimentin, and nestin which suppress barrier leakiness (Thanabalasundaram et al., 2011). Both TGF-β and bFGF are secreted from astrocytes (Abbott et al., 2006), further proving their role in regulating BBB functions, as well as interaction with ECs and pericytes. ECs specific TGF-β receptor III (Endoglin) the co-receptor of TGF-βRI, activates ALK1-Smad1/5/8, which can leads to vessel destabilization. In myofibroblast model, TGF-βRI activation may suppress Cav-1 expression via p38/MAPK pathway, and it’s shown to be independent to Smad activation (Sanders et al., 2015). This dual activity of TGF-β signaling should be investigated even further in BBB.

### Sphingosine-1-Phosphate (S1P)

Sphingosine-1-phosphate (S1P) is synthesized by two types of sphingosine kinase (Sphk 1 and 2). The HBMECs only expressed four out of five known S1P G-protein coupled receptors. S1P exposure to ECs might induce proangiogenic gene expression, cell migration, maintenance of cell proliferation, and inhibition of apoptosis (Kimura et al., 2001; Kuwabara et al., 2003). Secretion of S1P from pericytes and astrocytes to retinal microvasculature ECs will promote barrier stability through upregulation of various junctional proteins, as well as the expression of N-cadherin which promotes cell-to-cell interaction (Paik et al., 2004; McGuire et al., 2011). Expression of S1P will induce activation of the PI3K/protein kinase B (Akt/PKB) pathway and also upregulate antiapoptotic Bcl-2 and downregulate proapoptotic Bim (Limaye et al., 2005). Upregulation and dephosphorylation of the junctional molecule PECAM-1 was also observed in HUVECs (Limaye et al., 2005). S1P and LRP1 showed synergistic effects on chemotactic migration of HBMECs (Vézina et al., 2018). Vessel carrier effects showed by the chaperone HDL-associated ApoM may deliver S1P to the S1P1 and S1P3 receptors, promoting ECs proliferation, preventing apoptosis, and also improve barrier stability at the BBB (Galvani et al., 2015; Ruiz et al., 2017). However, S1P3 receptor activation in astrocytes isolated from mice shows that it might activates RhoA which induces inflammatory cytokines and S1P expression, indicating an autocrine loop which participates in BBB breakdown (Dusaban et al., 2017). In HUVECs and mice model, S1P/S1P1R activity possibly have a vasoprotective effects by regulating the amount of proinflammatory adhesion proteins (in this case ICAM-1) (Galvani et al., 2015). Activation of S1P1R signaling was reported to induce translocation of N-cadherin (making the bond between general ECs and pericytes stronger), and it has also been proposed that it alters the adhesive property of N-cadherin. This activity in general ECs gives rise to complex cellular communication via various ligands interacting with a single receptor, but activated through different pathways (Paik et al., 2004). Loss of the S1P1R will induce BBB leakiness (Yanagida et al., 2017). Meanwhile, activation of this receptor will also contribute to the synthesis and also recovery of rat fat-pad ECs glycocalyx, which mediates vascular robustness and adsorptive-mediated transcytosis (Zeng et al., 2015). Conversely, S1P2 receptor plays a role in suppressing the PI3K pathway which is activated via S1P1R. This inhibition is achieved through the coupling mechanism of Rho-dependent activation of PTEN phosphatase. Activation of these pathways will induce vascular permeability, promoting disruption of adherens junctions and stimulates stress fibers resulting in the leaky barrier (Sanchez et al., 2007). Interestingly, activation of the PI3K/Akt pathway by VEGF has been discovered to induce transcytosis via actin dynamics and Cav-1 activation (Wang et al., 2011; Jin et al., 2015; Chen et al., 2018). Multiple responses from PI3K/Akt pathway activation or suppression is indicating another regulatory pathway is necessary for a balanced transcytosis in ECs, and the outcome of this pathway may differ depending on the cell’s dynamics. A previous study using HeLa cells showed that S1P regulates transport proteins tetraspanins (CD63, CD81) and flotillin into exosomes in the process of MVEs (Multi Vesicular Endosomes) maturation (Kajimoto et al., 2013). S1P also has a protective effect on general ECs and adheren junctions, as well as actin and cytoskeleton arrangement (Kajimoto et al., 2013; Shepherd et al., 2017). In regulation of the synaptic system, sphingosine was shown to regulate the assembly of SNARE complex via synaptobrevin (Darios et al., 2009). In neurons, S1P also regulates localization of synapsin I, showing supporting activity of exocytosis process (Riganti et al., 2016). It is currently unknown whether S1P also plays similar role in the HBMECs or BBB complex.

### Angiopoietin/Tyrosine Kinase With Immunoglobulin-Like and EGF-Like Domains (Ang/TIE)

**FIGURE 3:**
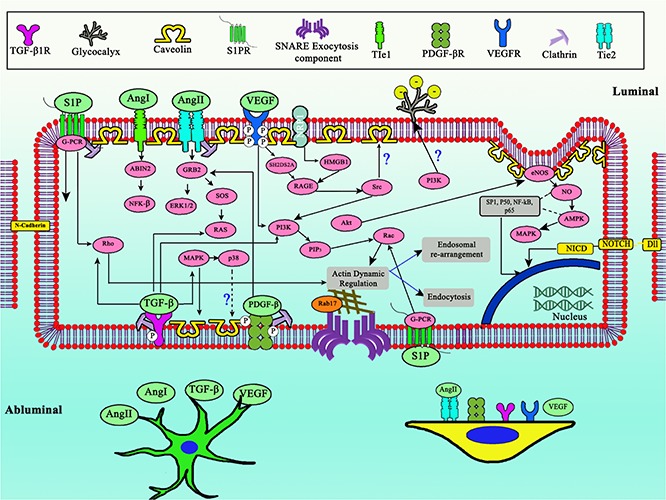
Complex shared developmental pathway between ECs, pericytes, and astrocytes to regulate transcytosis in BBB. Notice the central role of PI3K, updated model of TIE2 expressed in pericytes, as well as glycocalyx role for negative membrane charge. Dashed arrows indicates downregulation, straight line arrows indicates upregulation.

The mechanism of Ang/TIE pathway involves several angiopoietin ligands (Ang 1, 2, and 4 in humans) and TIE1/TIE2. Ang1 which is expressed from pericytes induces occludin expression in brain capillary ECs through TIE2 activation, thus promoting barrier tightness (Hori et al., 2004). Ang1 also inhibits FOXO1 activity via Akt activation in HUVECs, possibly interacting with various downstream target genes which involved in transcytosis (Daly et al., 2004). *In vitro* HUVECs study shows that after activation by Ang1, TIE2 will undergo internalization mediated by clathrin vesicles (Bogdanovic et al., 2009). Normally, Ang2 is not expressed in adult brain ECs, as Ang2 promotes barrier permeability via upregulation of Caveolin-1 (Cav-1) (Gurnik et al., 2016). Release of Ang2 showed dependency on VAMP3 in human brain ECs (Zhou et al., 2016). The expression of Ang1 and Ang2 will undergo changes during the normal aging process, whereby the former will be more expressed and meanwhile the latter will be suppressed. Expression of both receptors (TIE1 and TIE2) has been shown to be stable both in young ECs or adult ECs (Hohensinner et al., 2016). This regulation leads to vessel stability and ECs settlement in HUVECs (Hohensinner et al., 2016). Along with the senescence of HUVECs, some expression of junction proteins will be downregulated (Occludin and claudin-5), while ZO-1 will be upregulated compared to the younger ECs *in vitro* (Krouwer et al., 2012). A recent discovery has clarified that TIE2 receptors in human brain pericytes also play a vital role in the angiogenesis process (Teichert et al., 2017). Silencing of TIE2 expression in pericytes will induce pro-migratory phenotypes of ECs, indicating a close reciprocal relationship (Figure 3) between pericytes and ECs (Teichert et al., 2017). This discovery also provides a hypothetical connection between astrocytes and pericytes, since astrocytes also express Ang1 (Lee et al., 2003) which could regulate TIE2 in pericyte membranes.

### Notch

There are four types of Notch receptor in mammals, which showed interaction with five membrane-bound ligands, Jagged1, Jagged2, and also delta-like ligand (Dll) type 1, type 3, and type 4. Both brain pericytes and HUVECs showed expression of Jagged1 during co-culture (Kofler et al., 2015), indicating their importance in communication. Among several types of Notch receptors and ligands, only Dll4 and Notch4 specifically expressed on mammalian ECs (Shutter et al., 2000). In mice model, stimulation of Dll4 ligand will induce EphrinB2 expression in ECs. Furthermore, pericytes lacking in EphrinB2 expression will have a defects on vessel recruitment with ECs and impaired interaction with ECM (Foo et al., 2006). In a study using HUVECs, upregulation of Dll4 shows inhibition to the expression of VEGFR2 and NRP1, which regulates VEGF type A pathway (Williams et al., 2006), suggesting a mechanism to limit the number of caveolae and transcytosis across BBB. Inhibition of Notch signaling by GSI (γ-secretase inhibitor) showed its’ effects to increase blood vessel diameter, but not the vessel length, indicating a local shear stress regulation (Lee et al., 2017; Davis et al., 2018). Brain ECs showed activity to regulate astrocytes’ GLT-1 via Notch signaling pathway, which requires close contact between cells confirmed by *in vitro* experiment (Lee et al., 2017). This brings us to question- how does Notch signaling between ECs and astrocytes occur in the BBB when ECs are enveloped by pericytes and basal lamina? It has been discovered that Dll-4 and Jagged1 can be transported for intercellular communication, by passing through the extracellular matrix (Sheldon et al., 2010; Sharghi-Namini et al., 2014; Gonzalez-King et al., 2017). These discoveries bring our attention to the role of exosomes in signal trafficking, and that they possibly also regulate transcytosis.

## Factors Affecting Regulation of Transcytosis

**FIGURE 4:**
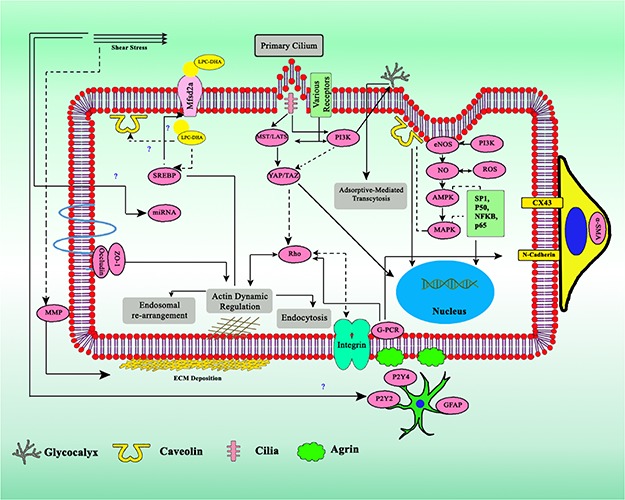
Crosstalk of various factors involved in the regulation of transcytosis. Biomechanics flow sensing by multiple mechanosensor gives several types of regulatory signal, together with miRNA, ECM, physicochemical factors, and various cytokines. Dashed arrows indicates downregulation, straight line arrows indicates upregulation.

Numerous factors might have a connection, either regulating directly or indirectly the transcytosis mechanism (Figure 4). In this review we would like to highlight some factors which have been indicated to regulate either endocytosis, endosomal rearrangement, exocytosis, or components of transcytosis.

### Physicochemical (pH, Temperature, O_2_, CO_2_, ROS) of the Molecules and Environment

Through several discoveries, physicochemical factors have been shown to play an indispensable role in balanced transcytosis in the BBB. Transcytosis via transferrin receptor showed a dependence on pH and the polarity of proteins (Sade et al., 2014). The release of iron also utilizes pH changes in endosomes to change the affinity between iron and transferrin (Qian et al., 2002). It is undetermined whether changes in pH might alter the expression of transcytosis’ components or not. A temperature shift induces membrane reorganization and actin dynamics which is cholesterol-dependent (Römer et al., 2010), indicating the role of temperature in governing transcytosis. Similar findings in neuron behavior highlights temperature-sensitive clathrin-independent endocytosis, which is mediated by dynamin and actin (Delvendahl et al., 2016). Further investigation is required to elucidate the mechanisms behind the effects of these factors. Oxygenation upregulates SSeCKS, a cytoskeleton protein which is expressed by astrocytes to invoke BBB tightness via VEGF suppression and Ang1 stimulation (Lee et al., 2003). Exposure to normobaric hyperoxia also can slow BBB damage (Liang et al., 2015). On the other hand, hyperoxia/ROS might induce the Fas-BID apoptosis signaling cascade, which is mediated by Cav-1 (Zhang et al., 2011). Signifying balanced regulation is necessary to maintain an appropriate amount of oxygen in the BBB. Hypoxic conditions might alter the content of exosomes for intercellular signaling. It has been shown that Notch ligands transport will be upregulated during hypoxia (Gonzalez-King et al., 2017). Hypoxia may induce oxidative stress, mainly caused by reperfusion (Thornton et al., 2017), triggering BBB breakdown via NOX4 activation (Casas et al., 2017). ROS which is produced by NOX is disruptive to the BBB, and has showed a dependence on cytokines, which actively downregulate junctional proteins in BBB (Rochfort et al., 2014). ROS disrupts brain ECs’ tight junctions arrangement via RhoA/PI3K/PKB pathway (Schreibelt et al., 2007). ROS also enhance the transcellular migration of monocytes across BBB (Van der Goes et al., 2002), possibly due to stimulation of caveolae production via c-Src (Coelho-Santos et al., 2016). H_2_O_2_ from ecSOD in caveolae might also promoting VEGF activity, which causing leakiness (Oshikawa et al., 2010). These data indicate that ROS will increase BBB permeability through transcytosis regulation. Supplementation of alpha lipoic acid (ALA) and melatonin helps to alleviate ECs oxidative stress brain (Patiño et al., 2016; Badran et al., 2018), possibly explored as the treatment.

### Mechanical Stress

The importance of normal blood flow for healthy brain microvasculature development since infancy has been proved (Farzam et al., 2017). Maintenance of regular blood flow by neurovascular control as well as cardiac function is prominent especially in childhood, and failure may lead to sleep-disordered breathing (Kontos et al., 2017). The maintenance of a healthy brain in adults is also closely related to normal hemodynamics, where individuals with cardiac problems will also suffer from brain aging (Sabayan et al., 2015). Hemodynamics affect neural activity and both systems are coupled and synchronized spatiotemporally, especially in excitatory neuron activity (Ma et al., 2016). One of the regulators between neural activity and HBMECs is pericytes, which control the capillary diameter within the central nervous system, depending on the neurotransmitter (Peppiatt et al., 2006). Pericyte activity as the regulator of blood flow in the neurovascular unit is also detected in the adult brain and during brain aging. Phenotypes such as BBB breakdown, neurodegeneration, and neuroinflammation were observed in pericyte-deficient model mice (Bell et al., 2010). *In vitro* experiments on bovine BMECs showed some proteins related to tight junction of BBB, Occludin and ZO-1 are regulated by blood flow (Berardi and Tarbell, 2009). When there is a higher shear stress, the expression will also be upregulated, and this process is dependent on cyclic strain (Collins et al., 2005). Mechanical stress has been shown to regulate cell behavior and other factors involving transcytosis. Shear stress affects the production of NO, independent of intracellular calcium (Chen et al., 2018). A recent discovery is that there is a close reciprocal connection between Hippo pathway (mechanosensory pathway) and caveolae. It has been elucidated that caveolae are regulating the mechanosensory action of cells, and they affect the expression of YAP/TAZ which is the transcription factor of Cav-1 and Cavin1 (Rausch et al., 2019). Shear stress also affects vessel growth by regulating miRNA expression (Guan et al., 2017). Reduced blood flow will alter ion homeostasis and receptor-mediated transcytosis of insulin at the BBB, but not significantly altered the paracellular transports (Hom et al., 2001). Pericyte ability to express α-SMA indicates the cell have a contractile ability for regulating blood vessel diameter and blood flow (Alarcon-martinez et al., 2018). In some study, effects of mechanical stress to the cell permeability has been well-elucidated. One example is in the renal epithelial cells, where fluid shear stress modulated the endocytosis via mTOR pathway (Long et al., 2017). In the HUVECs, shear stress affects the endocytosis through PECAM-1 via various pathways depending on the binding of distinct epitopes (Han et al., 2015).

It is indeed a result from specialization that HBMECs behave differently under exposure to shear stress compared to HUVECs as the representative of other ECs. HBMECs can maintain a cobblestone-like appearance under high shear stress, and most likely this mechanism is to minimize the paracellular transport by minimizing the length of tight junctions (Ye et al., 2014; Reinitz et al., 2015). The detailed explanation needs to be studied even further. In HBMECs, Mfsd2a (Major Facilitator Superfamily Domain Containing 2A) has been known to facilitate the uptake of DHA into brain (Nguyen et al., 2014) as well as maintaining low rates of transcytosis in the cerebrovascular units (Zhao and Zlokovic, 2014). By transporting DHA inside the cells, the caveolae vesicles formation can be inhibited by intracellular lipid concentration, thus promoting BBB integrity (Andreone et al., 2017). Mfsd2a expression is shown to be downregulated by the metastatic brain tumor to disrupt BBB integrity and lipid metabolism (Tiwary et al., 2018). Another study also showed a lethal microcephaly phenotype was shown in the absence of Mfsd2a (Guemez-gamboa et al., 2015). The expression of Mfsd2a showed partial dependency to LXR/Srebp1 and Srebp2 (Chan et al., 2018). Interestingly, shear stress was shown to activate Srebp1 splicing mediated by integrins in EC (Liu et al., 2002). SREBP splicing is also showed dependency to shear stress through S1P and S2P activation, allowing SREBP(N) to translocates into nucleus and activating SRE-mediated genes (Lin et al., 2003). Inferred from this pathway, this hypothesis opens up a possibility for explaining how mechanotransduction may affecting transcytosis. In astrocytes, mechanical stress is positively regulating the expression of GFAP. P2Y2 and P2Y4 are the mediators of calcium signaling in astrocytes, which also colocalize with GFAP (Paniagua-Herranz et al., 2017). These calcium receptors are dependent to caveolae regulation (Pani and Singh, 2009), suggesting a crosstalk between these factors to transcytosis process in astrocytes, as well as feedback regulation for promoting caveolae formation.

### Basement Membrane and Junctions Remodeling

Close contacts between neurovascular units are maintained through several ways. One of them is the peg-socket junctional complex, where the pericytes act like a peg and are inserted into EC’s sockets through facilitation of proteins, such as N-cadherin and connexin 43 (CX43) hemichannels. Hemichannels are membrane protein structures which are coupled to each other in adjacent cells, providing a channel for signaling molecules and exchange of metabolites (Orellana et al., 2011). The roles of CX43 as hemichannel between pericytes and ECs has been clearly elucidated. It has a crucial part in the maintenance of intercellular communication between pericytes and ECs, consequently promoting stability of barrier properties (Li and Roy, 2009; Bobbie et al., 2010). Inactivity of CX43 expression may leads to pericytes detachment and activation of ECs apoptosis (Tien et al., 2014). Gap junction alteration as part of tissue remodeling also contributes to alteration of transcytosis. During inflammation, expression of Cx43 and Cav-3 in astrocytes will be downregulated via iNOS activity. Cx43 as the regulator of gap junctions has also showed interaction with Cav-1/Cav-2 during transcytosis, however its relation with Cav-3 still has not been elucidated (Liao et al., 2010), leaving room for further study. N-cadherin is one of the transmembrane glycoproteins that is expressed by ECs, together with VE-cadherin. While VE-cadherin is indispensable for vascular morphogenesis, N-cadherin is essential to the process of vascular maturation through pericytes recruitment (Tillet et al., 2005). The regulation of N-cadherin is closely connected to the S1P pathway, where the activation of S1P1R will promote N-cadherin-dependent of the pericyte-EC connection (Paik et al., 2004). Albumin also plays a crucial role in the transcytosis of myeloperoxidase (MPO) via caveolae-albumin binding proteins (ABPs) (Tiruppathi et al., 2004). MPO itself will be localized at fibronectin and induce nitration of ECM, thus promoting the tissue remodeling by binding with adhesion plaques (Baldus et al., 2001). The expression of Cav-1 was upregulated after the induction of juvenile traumatic brain injury (jTBI), demonstrating signs of BBB repair attempts (Badaut et al., 2015). Upregulation of MMP-2 and MMP-9 expression was the result of the decreased amount of Cav-1, together with downregulation of TJ protein ZO-1. Rescue experiment using NOS inhibitor showed reserved expression of Cav-1, inhibition of MMPs activity, and restored BBB integrity (Gu et al., 2012; Sadeghian et al., 2018). Pericytes may induce rapid localized MMP-9 activity during ischemia (Underly et al., 2017). MMP-2 is the major contributor to occludin degeneration, meanwhile Cav-1 actively redistributes claudin-5 (Liu et al., 2012). BBB stabilization through astrocytic laminin (laminins-111 and -211) secretion occurs through pericytes’ integrin α2 (ITGA2) binding. Lack of astrocytic laminins may induce pericytes into contractile form, which compromise BBB integrity (Yao et al., 2014). Consequently, all these data represent exemplary cases of how basal lamina might regulate BBB transcytosis.

### Various Cytokines

The cytokine family contains the key molecules for cellular signaling. The exchange of cytokines between ECs, pericytes, and astrocytes are necessary to maintain BBB integrity, especially in the process of transcytosis (Dohgu and Banks, 2013). Discovery of the cytokines related to transcytosis is continuously studied, for example the CTRP5, which promotes LDL transcytosis (Li et al., 2018). It is indeed interesting that several pro-inflammatory cytokines are shown to be promoting transcytosis, one example is the HMGB1 which promotes albumin transcytosis through activation of Src and Cav-1 phosphorylation (Shang et al., 2016). Which bring us to question: what about dual-functioning cytokines? And also the anti-inflammatory cytokines?

Cytokine signaling has also showed a dependence on the transcytosis process. One case of this phenomenon is the trafficking of IL-11, which is known to maintain barrier function at the intestinal epithelium. Trafficking of IL-11 through IL-11R1 showed a unidirectional transcytosis process, and IL-11R1/2 also controls redirection of gp130 to the apical part of the cells (Monhasery et al., 2016). Following gp130, one of the inhibitors of the JAK/STAT pathway, the SOCS3 was showed to have another function in the stabilization of cavin-1. In return, cavin-1 also modulate SOCS3 ability to inhibit IL-6 signaling via cAMP (Williams et al., 2018). In this case we can see clearly how cytokines might alter cell-signaling processes, regulating transcytosis and at the same time was also showed dependency on the transcytosis process. Further investigation should be conducted regarding the way cytokines might alter endosomal rearrangement, including whether the quantity of cytokines exposure plays a part in transcytosis, and vice versa.

### miRNA Intercellular Transport (EC, Pericytes, Astrocytes)

miRNA is another factor which is both regulating and regulated by transcytosis. It has been proven to actively contribute in cell-to-cell communication, a process heavily relying on transcytosis, mainly through EVs. During diabetic complication located on the limb, miR-503 through the shedding of microparticles (MPs) is transferred from ECs to pericytes, resulting in pericyte detachment and increased vessel permeability (Caporali et al., 2015). Cav-1 downregulation by miR-192 is also observed in synovial tissue fibroblast-like cells (Li et al., 2017), as well as another miR-199a-5p targeting clathrin in cancer cells (Huang et al., 2017). It is plausible to see whether miRNA also regulates BBB permeability and transcytosis, given the specialization of HBMECs and the neurovascular unit. An attempt has been made to characterize miR-155 effects on HBMECs (Chen et al., 2015), further research should be arranged to see the combined interaction when co-cultured with astrocytes and pericytes, or *in vivo* study. Previous study showed pericytes capability to regulate vasculogenesis by secreting miRNA targeting Fli1 (Larsson et al., 2009). Astrocytes through EVs of cytokines (TNF-α and IL-1β) also regulates miR-501-3p which disrupts tight junction (Chaudhuri et al., 2018; Toyama et al., 2018). Another study showed miR-107, which endogenously expressed in ECs but also present in the cerebrospinal fluid, may protect BBB robustness from amyloid-beta (Liu et al., 2016), effects of miRNA also supports recovery after intracerebral hemorrhage (Xi et al., 2017). In contrast, there are also miRNA with BBB disrupting activities, for example miR-155 disrupting tight junction protein expression (Zheng et al., 2017), and miR-181c which secreted by cancer cells (Tominaga et al., 2015). There have been several findings where miRNA was alternating the course of signaling pathway related to BBB transcytosis, mainly VEGF (Chamorro-Jorganes et al., 2016), Ang/Tie (Fang et al., 2016), PDGFRβ (Tanaka et al., 2013), and TGF-beta (Zhou et al., 2016). miRNA was also showed to interact with other factors concerning transcytosis, especially mechanotransduction pathway (Demolli et al., 2015) and cytokines expression (Guo et al., 2017). Altogether, miRNA functions and transport system might be one of the factors affecting and also affected by transcytosis. Further research is needed to elucidate the detail mechanism and interaction in order to design an effective treatment strategy.

## Conclusion: Future Applications and Perspectives

By exploring factors of transcytosis which have been described above, we can apply the knowledge to the development of drug design and also BBB-on-a-chip.

### Drug Therapy Design

One of the obstacles for brain disease treatment is the special design and low permeability of the BBB which requires customized drug design. This design will enable drugs to be taken for transport, which mainly involves caveolae-dependent transcytosis (Choi et al., 2013; Piazzini et al., 2018). Several candidates have been tested as potential protagonists of Cav-1, which upregulates caveolae formation (Table 2). Nevertheless, specific protagonists of Cav-1 still have not been found, requiring future study. Targeting the growth factor receptor also seems promising for inducing caveolae formation, even though specificity and delivery should be considered. There are also agents targeting other receptors which may have an indirect effects to caveolae formation (Table 2). Targeting S1P1R might induce small molecule selective of BBB opening, indicating a possibility for drug administration (Yanagida et al., 2017). Recent study successfully create a temperature sensitive liposome (Bredlau et al., 2018) utilizing hyperthermia, and conjugated cation transporter which utilize ECs’ glycocalyx negative charge (Kou et al., 2018). Inactivating P-gp remains a challenge to prevent drug efflux, and a recent study showed it can be internalized due to ROS activation dependent on Cav-1 (Hoshi et al., 2019), consequently emphasizing the control of transcytosis upregulating factors to drug therapy strategies.

**TABLE 2:** Some signaling receptors, along with the characteristics of endocytosis, exocytosis (if available), protagonist, and antagonist molecules.

**Signaling pathway membrane receptors**	**Endocytosis characteristics**	**Exocytosis characteristics**	**Protagonist**	**Antagonist**	**Relation to phenotype observed**
TGF-βR I	Novel clathrin/caveolae dependent (He et al., 2015)		CTGF, SDC2 (Chang, 2016) TGF-β	SB431542 (Paonessa et al., 2019), A 83-01 (Yakoub and Sadek, 2018)	In homozygous KO mice, lethality was observed. Severe hemorrhage and abnormal vessel development was also present (Larsson et al., 2001)
VEGFR 1	Caveolae dependent (Celus et al., 2017)		VEGF-A;B	Sunitinib, Pazopanib, Axitinib (Musumeci et al., 2012)	Lack of this receptor induced by tamoxifen may cause increased angiogenesis, upregulation of VEGFR2 expression, but non-significant BBB Permeability. It also cause lethality in germline mice (Ho et al., 2012).
VEGFR 2	Caveolae dependent (Caliceti et al., 2014)		VEGF-A;C;D;E, Gremlin,	Monomeric Gremlin^*C141A*^, Sorafenib, Sunitinib, Pazopanib, Vandetanib, Axitinib (Musumeci et al., 2012)	Deficient of blood-island formation and vasculogenesis was observed in the KO mice (Shalaby et al., 1995), in heterozygous KO mice, angiogenesis was perturbed (Oladipupo et al., 2018)
S1P1R	Clathrin dependent (Reeves et al., 2016), Caveolae dependent (Ephstein et al., 2013)		Fingolimod (Quancard et al., 2012), Ponesimod (Bell et al., 2018)	CYM5442 (Kim et al., 2018), NIBR-0213 (Quancard et al., 2012), AD2900 (Song et al., 2017)	Knockout mice showed lethality and severe hemorrhage since infancy (Liu et al., 2000)
TIE2	Clathrin dependent (Bogdanovic et al., 2009) Caveolae dependent (Hossain et al., 2017)		ANG1, ANG4	ANG2, ANG3	Global deletion will cause lethality to mice embryo, pericytes specific deletion may cause developmental delay and abnormal vessel maturation (Teichert et al., 2017)
Dll4	Clathrin dependent (Sheldon et al., 2010)	Exosomal markers: LAMP1 TSG101 Rab5 (Sheldon et al., 2010)		Dll4-Fc (Sheldon et al., 2010), MEDI0639 (Jenkins et al., 2012)	Heterozygous deletion will induce arteriovenous malformation and haploinsufficient lethality in mice (Krebs et al., 2004)
Notch	Clathrin dependent (Meloty-Kapella et al., 2012)			Egfl7 (Nichol et al., 2010)	Lack of Notch1 in KO mice cause lethality (Conlon et al., 1995), meanwhile artery enlargement and vein underdevelopment was observed in heterozygous KO (Kim et al., 2008)
G Protein-Coupled Receptor (GPCR)	Clathrin dependent Caveolae dependent (Zhang and Kim, 2017)		GRI977143, kynurenic acid, 3-methoxycatechol	GDP-β-S	In GPR124 KO mice, embryonic lethality, with abnormality in CNS vascular and BBB was observed (Cullen et al., 2011)
EphrinB2	Clathrin dependent (Gaitanos et al., 2016)		EphA4	Dasatinib (Barquilla and Pasquale, 2014)	Blocking of EphrinB2 may inhibit angiogenesis in brain via VEGFR2 regulation (Sawamiphak et al., 2010), global deletion causes defective angiogenesis especially in the head region, and also lethality in embryonic mice (Wang et al., 1998).
PDGFR- β	EphB2-Caveolae dependent (Nakayama et al., 2013) EphB2-null-Clathrin dependent			Sorafenib, Sunitinib (Musumeci et al., 2012)	Knockout mice showed excessive bleeding, hypoplasia of vascular smooth muscle cells in larger vessel, and lack of pericytes in microvasculature (Hellström et al., 1999)
Cav-1	-		PPARγ, Pioglitazone (Werion et al., 2016) Chlorogenic acid derivatives (Lee et al., 2017)	Lovastatin and/or Celecoxib (Shimato et al., 2013) GGTI-286 Incadronate (Iguchi et al., 2006)	Knockout mice showed loss of Cav-2 expression, endocytosis defect, hyper-proliferation and abnormal vascular development (Razani et al., 2001)
Caveolae-mediated endocytosis	-			MβCD (Moriyama et al., 2017; Wu et al., 2019) Nystatin (Burger et al., 2011)	-
Clathrin-mediated endocytosis	-			Dynasore (Wu et al., 2019)	-

### BBB-on-a-Chip

**FIGURE 5:**
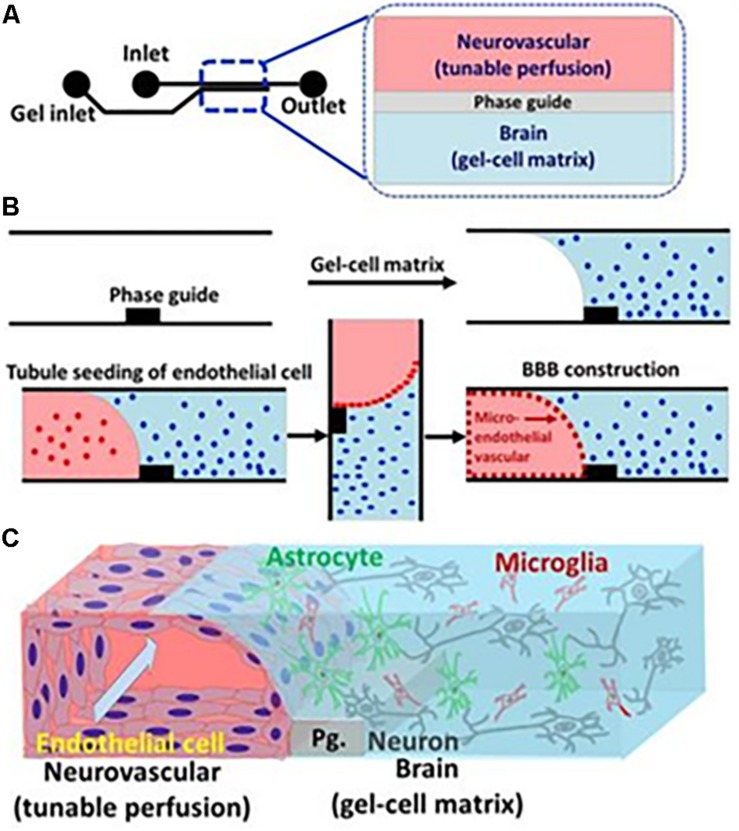
Illustration of BBBoC for drug or toxic compound testing. Interaction between various components of the BBB will regulate transcytosis resembling *in vivo* conditions. Reproduced with permission and courtesy of Yeoheung Yun (Koo et al., 2018). **(A)** Diagram of BBBoC design with inlet and outlet for medium flow. **(B)** Steps of cell seeding and gel-cell matrix formation. **(C)** Established model of BBBoC in this study.

The organ-on-a-chip offers a possibility to create a model closer to the human body than animal models and conventional cell culture models. One of the primary applications for BBBoC is the drug-testing field, to observe cytotoxicity and pharmacodynamics. It is also useful to study physiological interactions and responses from multiple organs (Ahadian et al., 2017). With relatively simple steps, and also time- and money-saving aspects, the organ-on-a-chip is the future model for experimentation and study models in the field of life science (Streets and Huang, 2013). Elucidation regarding transcytosis factors on the BBB will support establishment of BBBoC. For instance, a fruitful approach was taken by Koo et al. (2018), where a three-dimensional (3D) tetra-culture was made using combined construct of gel-cell matrix, phase guide, and perfusion of medium. The usage of phase guide which composed of capillary pressure barriers enable the separation of gel and fluid phases. Thus, the construction of membrane-free substrate for endothelial cell attachment was made possible, initiated by gel-cell polymerization which contained mix culture of microglia, astrocytes, and neuroblastoma combined to extracellular matrix that comprised of collagen. Cell seeding of ECs with perfused medium was done to mimics the shear stress and blood flow, which resulted in the development of neurovascular unit by ECs and gel-cell matrix (Figure 5). The permeability and integrity of BBBoC was tested by using AChE activity, viability, and residual organo phosphates (OPs) assay, which were known to be toxic and came across the brain through the BBB *in vivo*. Positive points of this model are the utilization of four types of cell which present *in vivo*, relatively normal permeability, and closely mimics the neurovascular unit (Koo et al., 2018). Some consideration that should be made is regarding the inability to measure TEER because of the difficulty to insert the electrodes, and another thing is the extracellular matrix which only comprised of collagen, whereas the *in vivo* extracellular matrix also comprised of fibronectin and gelatin. In conclusion, the field of BBBoC still has many possibilities for future development and integration to the body-on-a-chip system. BBBoC is a promising construct that may answer and serves as the future study model in many fields of life science.

Taken together, the whole study regarding factors of transcytosis in BBB still needs further exploration, especially regarding crosstalk between factors, context of environment and nutrition, as well as pathogenesis stimulation. With these factors in mind, application development will be more effective and efficient.

## Author Contributions

MT, YW, CD, and GW contributed conception and design of the study. NW and ZH organized the literature. VV performed the design of figures. MT wrote the first draft of the manuscript. All authors contributed to manuscript revision, read and approved the submitted version.

## Conflict of Interest

The authors declare that the research was conducted in the absence of any commercial or financial relationships that could be construed as a potential conflict of interest.

## References

[B1] Abbott N. J. Rönnbäck L. Hansson E. (2006). Astrocyte-endothelial interactions at the blood-brain barrier. *Nat. Rev. Neurosci.* 7 41–53. 10.1038/nrn1824 16371949

[B2] Ahadian S. Civitarese R. Bannerman D. Mohammadi M. H. Lu R. Wang E. (2017). Organ-on-a-chip platforms?: a convergence of advanced materials. cells, and microscale technologies. 7:1700506. 10.1002/adhm.201700506 29034591

[B3] Alarcon-martinez L. Yilmaz-ozcan S. Yemisci M. Schallek J. Kılç K. Can A. (2018). Capillary pericytes express α-smooth muscle actin, which requires prevention of filamentous-actin depolymerization for detection. *eLife* 7:e34861. 10.7554/eLife.34861 29561727 PMC5862523

[B4] Ando K. Tomimura K. Sazdovitch V. Suain V. Yilmaz Z. Authelet M. (2016). Level of PICALM, a key component of clathrin-mediated endocytosis, is correlated with levels of phosphotau and autophagy-related proteins and is associated with tau inclusions in AD. PSP and Pick disease. *Neurobiol. Dis.* 94 32–43. 10.1016/j.nbd.2016.05.017 27260836

[B5] Andreone B. J. Chow B. W. Tata A. Lacoste B. Ben-Zvi A. Bullock K. (2017). Blood-brain barrier permeability is regulated by lipid transport-dependent suppression of caveolae-mediated transcytosis. *Neuron* 94:581-594.e5. 10.1016/j.neuron.2017.03.043 28416077 PMC5474951

[B6] Argaw A. T. Asp L. Zhang J. Navrazhina K. Pham T. Mariani J. N. (2012). Astrocyte-derived VEGF-A drives blood-brain barrier disruption in CNS inflammatory disease. *J. Clin. Invest.* 122 2454–2468. 10.1172/JCI60842 22653056 PMC3386814

[B7] Armulik A. Genové G. Mäe M. Nisancioglu M. H. Wallgard E. Niaudet C. (2010). Pericytes regulate the blood-brain barrier. *Nature* 468 557–561. 10.1038/nature09522 20944627

[B8] Badaut J. Ajao D. O. Sorensen D. W. (2015). Caveolin expression changes in the neurovascular unit after juvenile traumatic brain injury?: signs of blood – brain barrier healing? *Neuroscience* 285 215–226. 10.1016/j.neuroscience.2014.10.035 25450954 PMC4431593

[B9] Badran M. Abuyassin B. H. Golbidi S. Ayas N. Laher I. (2018). Alpha lipoic acid improves endothelial function in mice subjected to chronic intermittent hypoxia. *Am. J. Respirat. Crit. Care Med.* 197:A3761. 10.1155/2019/4093018 PMC6481039 31093313

[B10] Bai X.-L. Yang X.-Y. Li J.-Y. Li Y. Jia X. Xiong Z.-F. (2017). Cavin-1 regulates caveolae-mediated LDL transcytosis: crosstalk in an AMPK/eNOS/NF-kB/Sp1 loop. *Oncotarget* 8 103985–103995. 10.18632/oncotarget.21944 29262615 PMC5732781

[B11] Bai Y. Zhu X. Chao J. Zhang Y. Qian C. Li P. (2015). Pericytes contribute to the disruption of the cerebral endothelial barrier via increasing VEGF expression?: implications for stroke. *PLoS One* 10:e0124362. 10.1371/journal.pone.0124362 25884837 PMC4401453

[B12] Baldus S. Eiserich J. P. Mani A. Castro L. Figueroa M. Chumley P. (2001). Endothelial transcytosis of myeloperoxidase confers specificity to vascular ECM proteins as targets of tyrosine nitration. *Clin. Chem.* 108 1759–1770. 10.1172/JCI200112617.Introduction 11748259 PMC209464

[B13] Barquilla A. Pasquale E. B. (2014). Eph receptors and ephrins: therapeutic opportunities. *Ann. Rev. Pharmacol. Toxicol.* 55 465–487. 10.1146/annurev-pharmtox-011112-140226 25292427 PMC4388660

[B14] Baumgartner I. Kalka C. Von Ballmoos M. W. Yang Z. Vo J. Santo S. D. (2010). Endothelial progenitor cells induce a phenotype shift in differentiated endothelial cells towards PDGF / PDGFR b axis-mediated angiogenesis. *PLoS One* 5:e0014107. 10.1371/journal.pone.0014107 21124835 PMC2991332

[B15] Bell M. Foley D. Naylor C. Robinson C. Riley J. Epemolu O. (2018). Discovery of super soft-drug modulators of sphingosine-1-phosphate receptor 1. *Bioorg. Med. Chem. Lett.* 28 3255–3259. 10.1016/j.bmcl.2018.07.044 30143424 PMC6185871

[B16] Bell R. D. Winkler E. A. Sagare A. P. Singh I. Larue B. Deane R. (2010). Article pericytes control key neurovascular functions and neuronal phenotype in the adult brain and during brain aging. *Neuron* 68 409–427. 10.1016/j.neuron.2010.09.043 21040844 PMC3056408

[B17] Berardi D. E. Tarbell J. M. (2009). Stretch and shear interactions affect intercellular junction protein expression and turnover in endothelial cells. *Cell Mol. Bioeng.* 2 320–331. 10.1007/s12195-009-0073-7 20161517 PMC2799298

[B18] Bobbie M. W. Roy S. Trudeau K. Munger S. J. Simon A. M. Roy S. (2010). Reduced connexin 43 expression and its effect on the development of vascular lesions in retinas of diabetic mice. *Invest. Ophthalmol. Vis. Sci.* 51 3758–3763. 10.1167/iovs.09-4489 20130277 PMC2904019

[B19] Bogdanovic E. Coombs N. Dumont D. J. (2009). Oligomerized Tie2 localizes to clathrin-coated pits in response to angiopoietin-1. *Histochem. Cell Biol.* 132 225–237. 10.1007/s00418-009-0603-3 19424712

[B20] Bredlau A. L. Motamarry A. Chen C. McCrackin M. A. Helke K. Armeson K. E. (2018). Localized delivery of therapeutic doxorubicin dose across the canine blood-brain barrier with hyperthermia and temperature sensitive liposomes. *Drug Deliv.* 25 973–984. 10.1080/10717544.2018.1461280 29688083 PMC6058514

[B21] Burger D. Montezano A. C. Nishigaki N. He Y. Carter A. Touyz R. M. (2011). Endothelial microparticle formation by angiotensin II is mediated via ang II receptor type I/NADPH Oxidase/rho kinase pathways targeted to lipid rafts. *Arteriosc. Thrombosis Vasc. Biol.* 31 1898–1907. 10.1161/ATVBAHA.110.222703 21597004

[B22] Cabezas R. Vega-Vela N. E. González-Sanmiguel J. González J. Esquinas P. Echeverria V. (2018). PDGF-BB preserves mitochondrial morphology, attenuates ros production, and upregulates neuroglobin in an astrocytic model under rotenone insult. *Mol. Neurobiol.* 55 3085–3095. 10.1007/s12035-017-0567-6 28466269

[B23] Caliceti C. Zambonin L. Rizzo B. Fiorentini D. Vieceli Dalla Sega F. Hrelia S. (2014). Role of plasma membrane caveolae/Lipid rafts in VEGF-induced redox signaling in human leukemia cells. *BioMed. Res. Int.* 2014 1–13. 10.1155/2014/857504 24738074 PMC3967716

[B24] Caporali A. Meloni M. Nailor A. Mitiæ T. Shantikumar S. Riu F. (2015). P75^*NTR*^-dependent activation of NF-κB regulates microRNA-503 transcription and pericyte-endothelial crosstalk in diabetes after limb ischaemia. *Nat. Commun.* 6:9024. 10.1038/ncomms9024 26268439 PMC4538859

[B25] Carroll S. J. O. Kho D. T. Wiltshire R. Nelson V. Rotimi O. Johnson R. (2015). Pro-inflammatory TNF α and IL-1 β differentially regulate the inflammatory phenotype of brain microvascular endothelial cells. *J. Neuroinflam.* 12:131. 10.1186/s12974-015-0346-0 26152369 PMC4506411

[B26] Casas A. I. Geuss E. Kleikers P. W. M. Mencl S. Herrmann A. M. Buendia I. (2017). NOX4-dependent neuronal autotoxicity and BBB breakdown explain the superior sensitivity of the brain to ischemic damage. *Proc. Natl. Acad. Sci. U.S.A.* 114 12315–12320. 10.1073/pnas.1705034114 29087944 PMC5699031

[B27] Celus W. Di Conza G. Oliveira A. I. Ehling M. Costa B. M. Wenes M. (2017). Loss of Caveolin-1 in metastasis-associated macrophages drives lung metastatic growth through increased angiogenesis. *Cell Rep.* 21 2842–2854. 10.1016/j.celrep.2017.11.034 29212030 PMC5732321

[B28] Cha S. H. Choi Y. R. Heo C. H. Kang S. J. Joe E. H. Jou I. (2015). Loss of parkin promotes lipid rafts-dependent endocytosis through accumulating caveolin-1: Implications for Parkinson’s disease. *Mol. Neurodegen.* 10 1–13. 10.1186/s13024-015-0060-5 26627850 PMC4666086

[B29] Chamorro-Jorganes A. Lee M. Y. Araldi E. Landskroner-Eiger S. Fernández-Fuertes M. Sahraei M. (2016). VEGF-induced expression of miR-17-92 cluster in endothelial cells is mediated by ERK/ELK1 activation and regulates angiogenesis. *Circ. Res.* 118 38–47. 10.1161/CIRCRESAHA.115.307408 26472816 PMC4703066

[B30] Chan J. P. Wong B. H. Chin C. F. Galam D. L. A. Foo J. C. Wong L. C. (2018). The lysolipid transporter Mfsd2a regulates lipogenesis in the developing brain. *PLoS Biol.* 16:e2006443. 10.1371/journal.pbio.2006443 30074985 PMC6093704

[B31] Chang C. (2016). Agonists and antagonists of the TGF-β family. *Cold Spring Harb. Perspect. Biol.* 8 203–258. 10.1101/cshperspect.a021923 PMC4968162 27413100

[B32] Chanthick C. Kanlaya R. Kiatbumrung R. Pattanakitsakul S. N. Thongboonkerd V. (2016). Caveolae-mediated albumin transcytosis is enhanced in dengue-infected human endothelial cells: a model of vascular leakage in dengue hemorrhagic fever. *Sci. Rep.* 6:31855. 10.1038/srep31855 27546060 PMC4992822

[B33] Chaudhuri A. D. Dastgheyb R. M. Yoo S. W. Trout A. Talbot C. C. Hao H. (2018). TNFα and IL-1β modify the miRNA cargo of astrocyte shed extracellular vesicles to regulate neurotrophic signaling in neurons article. *Cell Death Dis.* 9:363. 10.1038/s41419-018-0369-4 29507357 PMC5838212

[B34] Chen Y. Liu Y. Pan Q. Zhao Y. He C. Bi K. (2015). MicroRNA-155 regulates ROS production, NO generation, apoptosis and multiple functions of human brain microvessel endothelial cells under physiological and pathological conditions. *J. Cell. Biochem.* 116 2870–2881. 10.1002/jcb.25234 26012521

[B35] Chen Z. D’Arc Oliveira S. Zimnicka A. M. Jiang Y. Sharma T. Chen S. (2018). Reciprocal regulation of eNOS and Caveolin-1 functions in endothelial cells. *Mol. Biol. Cell* 29 1190–1202. 10.1091/mbc.e17-01-0049 29563255 PMC5935069

[B36] Choi C. H. J. Hao L. Narayan S. P. Auyeung E. Mirkin C. A. (2013). Mechanism for the endocytosis of spherical nucleic acid nanoparticle conjugates. *Proc. Natl. Acad. Sci. U.S.A.* 110 7625–7630. 10.1073/pnas.1305804110 23613589 PMC3651452

[B37] Chung J. W. Kim D. H. Oh M. J. Cho Y. H. Kim E. H. Moon G. J. (2018). Cav-1 (Caveolin-1) and arterial remodeling in adult moyamoya disease. *Stroke* 49 2597–2604. 10.1161/STROKEAHA.118.021888 30355208

[B38] Coelho-Santos V. Socodato R. Portugal C. Leitão R. A. Rito M. Barbosa M. (2016). Methylphenidate-triggered ROS generation promotes caveolae-mediated transcytosis via Rac1 signaling and c-Src-dependent caveolin-1 phosphorylation in human brain endothelial cells. *Cell. Mol. Life Sci.* 73 4701–4716. 10.1007/s00018-016-2301-3 27376435 PMC11108272

[B39] Collins N. T. Cummins P. M. Colgan O. C. Ferguson G. Birney Y. A. Murphy R. P. (2005). Cyclic strain – mediated regulation of vascular endothelial occludin and ZO-1 influence on intercellular tight junction assembly and function. *Arterioscler. Thromb. Vasc. Biol.* 26 62–68. 10.1161/01.ATV.0000194097.92824.b3 16269664

[B40] Conlon R. A. Reaume A. G. Rossant J. (1995). Notch1 is required for the coordinate segmentation of somites. *Development* 121 1533–1545. 7789282 10.1242/dev.121.5.1533

[B41] Cullen M. Elzarrad M. K. Seaman S. Zudaire E. Stevens J. Yang M. Y. (2011). GPR124, an orphan G protein-coupled receptor, is required for CNS-specific vascularization and establishment of the blood-brain barrier. *Proc. Natl. Acad. Sci. U.S.A.* 108 5759–5764. 10.1073/pnas.1017192108 21421844 PMC3078373

[B42] Daly A. C. Randall R. A. Hill C. S. (2008). Transforming growth factor ? -induced Smad1 / 5 phosphorylation in epithelial cells is mediated by novel receptor complexes and is essential for anchorage-independent growth. *Mol. Cell Biol.* 28 6889–6902. 10.1128/MCB.01192-08 18794361 PMC2573298

[B43] Daly C. Wong V. Burova E. Wei Y. Zabski S. Griffiths J. (2004). Angiopoietin-1 modulates endothelial cell function and gene expression via the transcription factor FKHR (FOXO1). *Genes Dev.* 18 1060–1071. 10.1101/gad.1189704 15132996 PMC406295

[B44] Darios F. Wasser C. Shakirzyanova A. Giniatullin A. Goodman K. Munoz-Bravo J. L. (2009). Sphingosine facilitates SNARE complex assembly and activates synaptic vesicle exocytosis. *Neuron* 62 683–694. 10.1016/j.neuron.2009.04.024 19524527 PMC2697323

[B45] Davis R. B. Pahl K. Datto N. C. Smith S. V. Shawber C. Caron K. M. (2018). Notch signaling pathway is a potential therapeutic target for extracranial vascular malformations. *Sci. Rep.* 8 1–10. 10.1038/s41598-018-36628-1 30573741 PMC6302123

[B46] Delvendahl I. Vyleta N. P. von Gersdorff H. Hallermann S. (2016). Fast, temperature-sensitive and clathrin-independent endocytosis at central synapses. *Neuron* 90 492–498. 10.1016/j.neuron.2016.03.013 27146271 PMC5125781

[B47] Demolli S. Doebele C. Doddaballapur A. Lang V. Fisslthaler B. Chavakis E. (2015). MicroRNA-30 mediates anti-inflammatory effects of shear stress and KLF2 via repression of angiopoietin 2. *J. Mol. Cell. Cardiol.* 88 111–119. 10.1016/j.yjmcc.2015.10.009 26456066

[B48] Dohgu S. Banks W. A. (2013). Brain pericytes increase the lipopolysaccharide-enhanced transcytosis of HIV-1 free virus across the in vitro blood – brain barrier?: evidence for cytokine-mediated pericyte-endothelial cell crosstalk. *Fluids Barr. CNS* 10:23. 10.1186/2045-8118-10-23 23816186 PMC3710206

[B49] Dusaban S. S. Chun J. Rosen H. Purcell N. H. Brown J. H. (2017). Sphingosine 1-phosphate receptor 3 and RhoA signaling mediate inflammatory gene expression in astrocytes. *J. Neuroinflam.* 14 1–10. 10.1186/s12974-017-0882-x 28577576 PMC5455202

[B50] Ephstein Y. Singleton P. A. Chen W. Wang L. Salgia R. Kanteti P. (2013). Critical role of S1PR1 and integrin β4 in HGF/c-Met-mediated increases in vascular integrity. *J. Biol. Chem.* 288 2191–2200. 10.1074/jbc.M112.404780 23212923 PMC3554892

[B51] Fang Z. He Q. W. Li Q. Chen X. L. Baral S. Jin H. J. (2016). MicroRNA-150 regulates blood-brain barrier permeability via Tie-2 after permanent middle cerebral artery occlusion in rats. *FASEB J.* 30 2097–2107. 10.1096/fj.201500126 26887441

[B52] Farg M. A. Sundaramoorthy V. Sultana J. M. Yang S. Atkinson R. A. K. Levina V. (2014). C9ORF72, implicated in amytrophic lateral sclerosis and frontotemporal dementia, regulates endosomal trafficking. *Hum. Mol. Genet.* 23 3579–3595. 10.1093/hmg/ddu068 24549040 PMC4049310

[B53] Farzam P. Buckley E. M. Lin P. Hagan K. Grant P. E. Inder T. E. (2017). Shedding light on the neonatal brain?: probing cerebral hemodynamics by diffuse optical spectroscopic methods. *Sci. Rep.* 8:6007. 10.1038/s41598-017-15995-1 29651161 PMC5897551

[B54] Ferrara N. Gerber H. Lecouter J. (2003). The biology of VEGF and its receptors. *Nat. Med.* 9 669–676. 10.1038/nm0603-669 12778165

[B55] Ferrari G. Cook B. D. Terushkin V. Pintucci G. Mignatti P. (2009). Transforming growth angiogenesis through vascular endothelial growth factor (VEGF) -mediated apoptosis. *J. Cell Physiol.* 219 449–458. 10.1002/jcp.21706 19180561 PMC2749291

[B56] Figley C. R. Stroman P. W. (2011). The role(s) of astrocytes and astrocyte activity in neurometabolism, neurovascular coupling, and the production of functional neuroimaging signals. *Eur. J. Neurosci.* 33 577–588. 10.1111/j.1460-9568.2010.07584.x 21314846

[B57] Foo S. S. Turner C. J. Adams S. Compagni A. Aubyn D. Kogata N. (2006). Ephrin-B2 controls cell motility and adhesion during blood-vessel-wall assembly. *Cell* 124 161–173. 10.1016/j.cell.2005.10.034 16413489

[B58] Gaitanos T. N. Koerner J. Klein R. (2016). Tiam-Rac signaling mediates trans-endocytosis of ephrin receptor EphB2 and is important for cell repulsion. *J. Cell Biol.* 214 1–18. 10.1083/jcb.201512010 27597758 PMC5021091

[B59] Gallini R. Lindblom P. Bondjers C. Betsholtz C. Andrae J. (2016). PDGF-A and PDGF-B induces cardiac fi brosis in transgenic mice. *Exp. Cell Res.* 349 282–290. 10.1016/j.yexcr.2016.10.022 27816607

[B60] Galvani S. Sanson M. Blaho V. A. Swendeman S. L. Conger H. Dahlbäck B. (2015). HDL-bound sphingosine 1-phosphate acts as a biased agonist for the endothelial cell receptor S1P 1 to limit vascular inflammation. *Sci. Signal.* 8 1–11. 10.1126/scisignal.aaa2581 26268607 PMC4768813

[B61] Gaudreault S. B. Dea D. Poirier J. (2004). Increased caveolin-1 expression in Alzheimer’s disease brain. *Neurobiol. Aging* 25 753–759. 10.1016/j.neurobiolaging.2003.07.004 15165700

[B62] Gonzalez-King H. García N. A. Ontoria-Oviedo I. Ciria M. Montero J. A. Sepúlveda P. (2017). Hypoxia inducible factor-1α potentiates jagged 1-mediated angiogenesis by mesenchymal stem cell-derived exosomes. *Stem Cells* 35 1747–1759. 10.1002/stem.2618 28376567

[B63] Gu Y. Zheng G. Xu M. Li Y. Chen X. Zhu W. (2012). Caveolin-1 regulates nitric oxide-mediated matrix metalloproteinases activity and blood–brain barrier permeability in focal cerebral ischemia and reperfusion injury. *J. Neurochem.* 120 147–156. 10.1111/j.1471-4159.2011.07542.x 22007835

[B64] Guan Y. Cai B. Wu X. Peng S. Gan L. Huang D. (2017). MicroRNA-352 regulates collateral vessel growth induced by elevated fluid shear stress in the rat hind limb. *Sci. Rep.* 7 1–13. 10.1038/s41598-017-06910-9 28751690 PMC5532297

[B65] Guemez-gamboa A. Nguyen L. N. Yang H. Zaki M. S. Kara M. Ben-omran T. (2015). Inactivating mutations in MFSD2A, required for omega-3 fatty acid transport in brain, cause a lethal microcephaly syndrome. *Nat. Genet.* 47 809–813. 10.1038/ng.3311 26005868 PMC4547531

[B66] Guo J. Li J. Zhao J. Yang S. Wang L. Cheng G. (2017). MiRNA-29c regulates the expression of inflammatory cytokines in diabetic nephropathy by targeting tristetraprolin. *Sci. Rep.* 7 1–13. 10.1038/s41598-017-01027-5 28539664 PMC5443806

[B67] Gurnik S. Devraj K. Macas J. Yamaji M. Starke J. Scholz A. (2016). Angiopoietin - 2 - induced blood – brain barrier compromise and increased stroke size are rescued by VE - PTP - dependent restoration of Tie2 signaling. *Acta Neuropathol.* 131 753–773. 10.1007/s00401-016-1551-3 26932603 PMC4835530

[B68] Han J. Shuvaev V. V. Davies P. F. Eckmann D. M. Muro S. Muzykantov V. R. (2015). Flow shear stress differentially regulates endothelial uptake of nanocarriers targeted to distinct epitopes of PECAM-1. *J. Control. Release* 210 39–47. 10.1016/j.jconrel.2015.05.006 25966362 PMC4793278

[B69] Hayer A. Stoeber M. Bissig C. Helenius A. (2010). Biogenesis of caveolae: Stepwise assembly of large caveolin and cavin complexes. *Traffic* 11 361–382. 10.1111/j.1600-0854.2009.01023.x 20070607

[B70] He K. Yan X. Li N. Dang S. Xu L. Zhao B. (2015). Internalization of the TGF-β type i receptor into caveolin-1 and EEA1 double-positive early endosomes. *Cell Res.* 25 738–752. 10.1038/cr.2015.60 25998683 PMC4456627

[B71] Head B. P. Peart J. N. Panneerselvam M. Yokoyama T. Pearn M. L. Niesman I. R. (2010). Loss of caveolin-1 accelerates neurodegeneration and aging. *PLoS One* 5:e0015697. 10.1371/journal.pone.0015697 21203469 PMC3009734

[B72] Hellström M. Kalén M. Lindahl P. Abramsson A. Betsholtz C. (1999). Role of PDGF-B and PDGFR- β in recruitment of vascular smooth muscle cells and pericytes during embryonic blood vessel formation in the mouse. *Development* 126 3047–3055. 10375497 10.1242/dev.126.14.3047

[B73] Hergenreider E. Heydt S. Tréguer K. Boettger T. Horrevoets A. J. G. Zeiher A. M. (2012). Atheroprotective communication between endothelial cells and smooth muscle cells through miRNAs. *Nat. Publish. Group* 14 249–256. 10.1038/ncb2441 22327366

[B74] Hirama T. Das R. Yang Y. Ferguson C. Won A. Yip C. M. (2017). Phosphatidylserine dictates the assembly and dynamics of caveolae in the plasma membrane. *J. Biol. Chem.* 292 14292–14307. 10.1074/jbc.M117.791400 28698382 PMC5572903

[B75] Ho V. C. Duan L. J. Cronin C. Liang B. T. Fong G. H. (2012). Elevated vascular endothelial growth factor receptor-2 abundance contributes to increased angiogenesis in vascular endothelial growth factor receptor-1-deficient mice. *Circulation* 126 741–752. 10.1161/CIRCULATIONAHA.112.091603 22753193 PMC3442373

[B76] Hohensinner P. J. Ebenbauer B. Kaun C. Maurer G. Huber K. Wojta J. (2016). Reduced Ang2 expression in aging endothelial cells. *Biochem. Biophys. Res. Commun.* 474 447–451. 10.1016/j.bbrc.2016.04.143 27137842

[B77] Hom S. Egleton R. D. Huber J. D. Davis T. P. (2001). Effect of reduced flow on blood – brain barrier transport systems. *Brain Res.* 890 38–48. 10.1016/s0006-8993(00)03027-4 11164767

[B78] Hori S. Ohtsuki S. Hosoya K. Nakashima E. (2004). A pericyte-derived angiopoietin-1 multimeric complex induces occludin gene expression in brain capillary endothelial cells through Tie-2 activation in vitro. *J. Neurochem.* 89 503–513. 10.1111/j.1471-4159.2004.02343.x 15056293

[B79] Hoshi Y. Uchida Y. Tachikawa M. Ohtsuki S. Couraud P. O. Suzuki T. (2019). Oxidative stress-induced activation of Abl and Src kinases rapidly induces P-glycoprotein internalization via phosphorylation of caveolin-1 on tyrosine-14, decreasing cortisol efflux at the blood–brain barrier. *J. Cereb. Blood Flow Metab.* 10.1177/0271678X18822801 [Epub ahead of print]. 30621530 PMC7370610

[B80] Hossain M. B. Shifat R. Li J. Luo X. Hess K. R. Rivera-Molina Y. (2017). TIE2 associates with caveolae and regulates caveolin-1 to promote their nuclear translocation. *Mol. Cell. Biol.* 37:e142-17. 10.1128/mcb.00142-17 28760776 PMC5640814

[B81] Huang G. H. Shan H. Li D. Zhou B. Pang P. F. (2017). MiR-199a-5p suppresses tumorigenesis by targeting clathrin heavy chain in hepatocellular carcinoma. *Cell Biochem. Funct.* 35 98–104. 10.1002/cbf.3252 28261837

[B82] Iguchi K. Matsunaga S. Nakano T. Usui S. Hirano K. (2006). Inhibition of caveolin-1 expression by incadronate in PC-3 prostate cells. *Anticancer Res.* 26 2977–2981. 16886623

[B83] Imakita N. Kitabatake M. Ouji-Sageshima N. Hara A. Morita-Takemura S. Kasahara K. (2019). Abrogated Caveolin-1 expression via histone modification enzyme Setdb2 regulates brain edema in a mouse model of influenza-associated encephalopathy. *Sci. Rep.* 9 1–12. 10.1038/s41598-018-36489-8 30670717 PMC6342998

[B84] Jenkins D. W. Ross S. Veldman-Jones M. Foltz I. N. Clavette B. C. Manchulenko K. (2012). MEDI0639: a novel therapeutic antibody targeting Dll4 modulates endothelial cell function and angiogenesis in vivo. *Mol. Cancer Therapeut.* 11 1650–1660. 10.1158/1535-7163.mct-11-1027 22679110

[B85] Jin J. Peng C. Wu S. Z. Chen H. M. Zhang B. F. (2015). Blocking VEGF/Caveolin-1 signaling contributes to renal protection of fasudil in streptozotocin-induced diabetic rats. *Acta Pharmacol. Sinica* 36 831–840. 10.1038/aps.2015.23 25937636 PMC4648118

[B86] Kajimoto T. Okada T. Miya S. Zhang L. Nakamura S. I. (2013). Ongoing activation of sphingosine 1-phosphate receptors mediates maturation of exosomal multivesicular endosomes. *Nat. Commun.* 4 1–13. 10.1038/ncomms3712 24231649

[B87] Kawamata H. Ng S. K. Diaz N. Burstein S. Morel L. Osgood A. (2014). Abnormal intracellular calcium signaling and SNARE-dependent exocytosis contributes to SOD1G93A astrocyte-mediated toxicity in amyotrophic lateral sclerosis. *J. Neurosci.* 34 2331–2348. 10.1523/jneurosci.2689-13.2014 24501372 PMC3913875

[B88] Kawedia J. D. Nieman M. L. Boivin G. P. Melvin J. E. Kikuchi K.-I. Hand A. R. (2007). Interaction between transcellular and paracellular water transport pathways through Aquaporin 5 and the tight junction complex. *Proc. Natl. Acad. Sci. U.S.A.* 104 3621–3626. 10.1073/pnas.0608384104 17360692 PMC1802728

[B89] Kim S. J. Bielawski J. Yang H. Kong Y. Zhou B. Li J. (2018). Functional antagonism of sphingosine-1-phosphate receptor 1 prevents cuprizone-induced demyelination. *Glia* 66 654–669. 10.1002/glia.23272 29193293 PMC5773114

[B90] Kim Y. H. Hu H. Guevara-Gallardo S. Lam M. T. Y. Fong S. Y. Wang R. A. (2008). Artery and vein size is balanced by Notch and ephrin B2/EphB4 during angiogenesis. *Development* 135 3755–3764. 10.1242/dev.022475 18952909 PMC2596923

[B91] Kimura T. Sato K. Kuwabara A. Tomura H. Ishiwara M. Kobayashi I. (2001). Sphingosine 1-phosphate may be a major component of plasma lipoproteins responsible for the cytoprotective actions in human umbilical vein endothelial cells. *J. Biol. Chem.* 276 31780–31785. 10.1074/jbc.M104353200 11427538

[B92] Knowland D. Arac A. Sekiguchi K. J. Hsu M. Lutz S. E. Perrino J. (2014). Stepwise recruitment of transcellular and paracellular pathways underlies blood-brain barrier breakdown in stroke. *Neuron* 82 603–617. 10.1016/j.neuron.2014.03.003 24746419 PMC4016169

[B93] Kofler N. M. Cuervo H. Uh M. K. Murtomäki A. Kitajewski J. (2015). *Combined Deficiency of Notch1 and Notch3 Causes Pericyte Dysfunction, Models CADASIL, and Results in Arteriovenous Malformations.* Berlin: Nature Publishing Group, 1–13. 10.1038/srep16449 PMC4643246 26563570

[B94] Kontos A. Lushington K. Martin J. Schwarz Q. Green R. Wabnitz D. (2017). Relationship between vascular resistance and sympathetic nerve fiber density in arterial vessels in children with sleep disordered breathing. *J. Am. Heart Assoc.* 6 1–11. 10.1161/JAHA.117.006137 28716800 PMC5586314

[B95] Koo Y. Hawkins B. T. Yun Y. (2018). Three-dimensional (3D) tetra-culture brain on chip platform for organophosphate toxicity screening. *Sci. Rep.* 8 1–7. 10.1038/s41598-018-20876-2 29434277 PMC5809488

[B96] Kou L. Hou Y. Yao Q. Guo W. Wang G. Wang M. (2018). L-Carnitine-conjugated nanoparticles to promote permeation across blood–brain barrier and to target glioma cells for drug delivery via the novel organic cation/carnitine transporter OCTN2. *Artif. Cells Nanomed. Biotechnol.* 46 1605–1616. 10.1080/21691401.2017.1384385 28974108

[B97] Krebs L. T. Shutter J. R. Tanigaki K. Honjo T. Stark K. L. Gridley T. (2004). Haploinsufficient lethality and formation of arteriovenous malformations in Notch pathway mutants. *Genes Dev.* 18 2469–2473. 10.1101/gad.1239204 15466160 PMC529533

[B98] Krouwer V. J. D. Hekking L. H. P. Langelaar-Makkinje M. Regan-Klapisz E. Post J. A. (2012). Endothelial cell senescence is associated with disrupted cell-cell junctions and increased monolayer permeability. *Vasc. Cell* 4 1–10. 10.1186/2045-824X-4-12 22929066 PMC3527188

[B99] Kuan W. L. Bennett N. He X. Skepper J. N. Martynyuk N. Wijeyekoon R. (2016). α-Synuclein pre-formed fibrils impair tight junction protein expression without affecting cerebral endothelial cell function. *Exp. Neurol.* 285 72–81. 10.1016/j.expneurol.2016.09.003 27632900

[B100] Kuwabara A. Murakami M. Okajima F. (2003). High-density lipoprotein stimulates endothelial cell migration and survival through sphingosine 1-phosphate and its receptors. *Arterioscler. Thromb. Vasc. Biol.* 23 1283–1288. 10.1161/01.ATV.0000079011.67194.5A 12775579

[B101] Lalatsa A. Butt A. M. (2018). “Physiology of the blood–brain barrier and mechanisms of transport across the BBB,” in *Nanotechnology-Based Targeted Drug Delivery Systems for Brain Tumors*, eds Kesharwani P. Gupta U. (Cambridge, MA: Academic Press), 10.1016/b978-0-12-812218-1.00003-8

[B102] Larsson E. Fuchs P. F. Heldin J. Barkefors I. Bondjers C. Genové G. (2009). Discovery of microvascular miRNAs using public gene expression data: MiR-145 is expressed in pericytes and is a regulator of Fli1. *Genome Med.* 1 1–12. 10.1186/gm108 19917099 PMC2808743

[B103] Larsson J. Goumans M. J. Sjöstrand L. J. Van Rooijen M. A. Ward D. Levéen P. (2001). Abnormal angiogenesis but intact hematopoietic potential in TGF-β type I receptor-deficient mice. *EMBO J.* 20 1663–1673. 10.1093/emboj/20.7.1663 11285230 PMC145465

[B104] Lee M. L. Martinez-Lozada Z. Krizman E. N. Robinson M. B. (2017). Brain endothelial cells induce astrocytic expression of the glutamate transporter GLT-1 by a Notch-dependent mechanism. *J. Neurochem.* 143 489–506. 10.1111/jnc.14135 28771710 PMC5693650

[B105] Lee S. W. Kim W. J. Choi Y. K. Song H. S. Son M. J. Gelman I. H. (2003). SSeCKS regulates angiogenesis and tight junction formation in blood-brain barrier. *Nat. Med.* 9 900–906. 10.1038/nm889 12808449

[B106] Lee Y. J. Hsu J. D. Lin W. L. Kao S. H. Wang C. J. (2017). Upregulation of caveolin-1 by mulberry leaf extract and its major components, chlorogenic acid derivatives, attenuates alcoholic steatohepatitis: via inhibition of oxidative stress. *Food Funct.* 8 497–405. 10.1039/c6fo01539e 28074952

[B107] Lengfeld J. E. Lutz S. E. Smith J. R. Diaconu C. Scott C. Kofman S. B. (2017). Endothelial Wnt/β-catenin signaling reduces immune cell infiltration in multiple sclerosis. *Proc. Natl. Acad. Sci. U.S.A.* 114 E1168–E1177. 10.1073/pnas.1609905114 28137846 PMC5320985

[B108] Li A. F. Roy S. (2009). High glucose-induced downregulation of connexin 43 expression promotes apoptosis in microvascular endothelial cells. *Invest. Ophthalmol. Vis. Sci.* 50 1400–1407. 10.1167/iovs.07-1519 19029021

[B109] Li C. Chen J. W. Liu Z. H. Shen Y. Ding F. H. Gu G. (2018). CTRP5 promotes transcytosis and oxidative modification of low-density lipoprotein and the development of atherosclerosis. *Atherosclerosis* 278 197–209. 10.1016/j.atherosclerosis.2018.09.037 30300788

[B110] Li F. Lan Y. Wang Y. Wang J. Yang G. Meng F. (2011). Endothelial Smad4 maintains cerebrovascular integrity by activating N-Cadherin through cooperation with Notch. *Dev. Cell* 20 291–302. 10.1016/j.devcel.2011.01.011 21397841

[B111] Li S. Jin Z. Lu X. (2017). MicroRNA-192 suppresses cell proliferation and induces apoptosis in human rheumatoid arthritis fibroblast-like synoviocytes by downregulating caveolin 1. *Mol. Cell. Biochem.* 432 123–130. 10.1007/s11010-017-3003-3 28321538

[B112] Liang J. Qi Z. Liu W. Wang P. Shi W. Dong W. (2015). Normobaric hyperoxia slows blood-brain barrier damage and expands the therapeutic time window for tissue-type plasminogen activator treatment in cerebral ischemia. *Stroke* 46 1344–1351. 10.1161/strokeaha.114.008599 25804925 PMC4414814

[B113] Liao C. K. Wang S. M. Chen Y. L. Wang H. S. Wu J. C. (2010). Lipopolysaccharide-induced inhibition of connexin43 gap junction communication in astrocytes is mediated by downregulation of caveolin-3. *Int. J. Biochem. Cell Biol.* 42 762–770. 10.1016/j.biocel.2010.01.016 20093193

[B114] Lim J. C. Kania K. D. Wijesuriya H. Chawla S. Sethi J. K. Pulaski L. (2008). Activation of β-catenin signalling by GSK-3 inhibition increases p-glycoprotein expression in brain endothelial cells. *J. Neurochem.* 106 1855–1865. 10.1111/j.1471-4159.2008.05537.x 18624906 PMC4303914

[B115] Limaye V. Li X. Hahn C. Xia P. Berndt M. C. Vadas M. A. (2005). Sphingosine kinase-1 enhances endothelial cell survival through a PECAM-1-dependent activation of PI-3K/Akt and regulation of Bcl-2 family members. *Blood* 105 3169–3177. 10.1182/blood-2004-02-0452 15632208

[B116] Lin T. Zeng L. Liu Y. DeFea K. Schwartz M. A. Chien S. (2003). Rho-ROCK-LIMK-cofilin pathway regulates shear stress activation of sterol regulatory element binding proteins. *Circ. Res.* 92 1296–1304. 10.1161/01.RES.0000078780.65824.8B 12775580

[B117] Lindahl P. Johansson B. R. Levéen P. Betsholtz C. (1997). Pericyte loss and microaneurysm formation in PDGF-B-deficient mice. *Science* 277 242–245. 10.1126/science.277.5323.242 9211853

[B118] Liu J. Jin X. Liu K. J. Liu W. (2012). Matrix metalloproteinase-2-mediated occludin degradation and caveolin-1-mediated claudin-5 redistribution contribute to blood-brain barrier damage in early ischemic stroke stage. *J. Neurosci.* 32 3044–3057. 10.1523/JNEUROSCI.6409-11.2012 22378877 PMC3339570

[B119] Liu W. Cai H. Lin M. Zhu L. Gao L. Zhong R. (2016). MicroRNA-107 prevents amyloid-beta induced blood-brain barrier disruption and endothelial cell dysfunction by targeting Endophilin-1. *Exp. Cell Res.* 343 248–257. 10.1016/j.yexcr.2016.03.026 27038654

[B120] Liu Y. Chen B. P. C. Lu M. Zhu Y. Stemerman M. B. Chien S. (2002). Shear stress activation of SREBP1 in endothelial cells is mediated by integrins. *Arterioscler. Thromb. Vasc. Biol.* 22 76–81. 10.1161/hq0102.101822 11788464

[B121] Liu Y. Wada R. Yamashita T. Mi Y. Deng C. X. Hobson J. P. (2000). Edg-1, the G protein-coupled receptor for sphingosine-1-phosphate, is essential for vascular maturation. *J. Clin. Invest.* 106 951–961. 10.1172/JCI10905 11032855 PMC314347

[B122] Long K. R. Shipman K. E. Rbaibi Y. Menshikova E. V. Ritov V. B. Eshbach M. L. (2017). Proximal tubule apical endocytosis is modulated by fluid shear stress via an mTOR-dependent pathway. *Mol. Biol. Cell* 28 2508–2517. 10.1091/mbc.e17-04-0211 28720662 PMC5597323

[B123] Lutz S. E. Smith J. R. Kim D. H. Olson C. V. L. Ellefsen K. Bates J. M. (2017). Caveolin1 is required for Th1 cell infiltration, but not tight junction remodeling, at the blood-brain barrier in autoimmune neuroinflammation. *Cell Rep.* 21 2104–2117. 10.1016/j.celrep.2017.10.094 29166603 PMC5728697

[B124] Ma Y. Shaik M. A. Kozberg M. G. Kim S. H. Portes J. P. Timerman D. (2016). Resting-state hemodynamics are spatiotemporally coupled to synchronized and symmetric neural activity in excitatory neurons. *Proc. Natl. Acad. Sci. U.S.A.* 113 E8463–E8471. 10.1073/pnas.1525369113 27974609 PMC5206542

[B125] Ma Y. Zhang Z. Chen R. Shi R. Zeng P. Chen R. (2019). NRP1 regulates HMGB1 in vascular endothelial cells under high homocysteine condition. *Am. J. Physiol. Heart Circ. Physiol.* 316 H1039–H1046. 10.1152/ajpheart.00746.2018 30767669

[B126] Marcu R. Choi Y. J. Xue J. Stephen M. Marcu R. Choi Y. J. (2018). Human organ-specific endothelial cell heterogeneity human organ-specific endothelial cell heterogeneity. *IScience* 4 20–35. 10.1016/j.isci.2018.05.003 30240741 PMC6147238

[B127] Mayhan W. (1999). VEGF increases permeability of the blood-brain barrier via a nitric oxide synthase/cGMP-dependent pathway. *Am. J. Physiol. Cell* 276 C1148–C1153. 10.1152/ajpcell.1999.276.5.C1148 10329964

[B128] McGuire P. G. Rangasamy S. Maestas J. Das A. (2011). Pericyte-derived sphinogosine 1-phosphate induces the expression of adhesion proteins and modulates the retinal endothelial cell barrier. *Arterioscler. Thromb. Vasc. Biol.* 31 107–115. 10.1161/ATVBAHA.111.235408 21940944 PMC3225006

[B129] Meloty-Kapella L. Shergill B. Kuon J. Botvinick E. Weinmaster G. (2012). Notch ligand endocytosis generates mechanical pulling force dependent on dynamin. epsins, and actin. *Dev. Cell* 22 1299–1312. 10.1016/j.devcel.2012.04.005 22658936 PMC3400432

[B130] Monhasery N. Moll J. Cuman C. Franke M. Lamertz L. Nitz R. (2016). transcytosis of IL-11 and apical redirection of gp130 is mediated by IL-11α receptor. *Cell Rep.* 16 1067–1081. 10.1016/j.celrep.2016.06.062 27425614

[B131] Moriyama T. Sasaki K. Karasawa K. Uchida K. Nitta K. (2017). Intracellular transcytosis of albumin in glomerular endothelial cells after endocytosis through caveolae. *J. Cell. Physiol.* 232 3565–3573. 10.1002/jcp.25817 28112392

[B132] Muhl L. Folestad E. B. Gladh H. Wang Y. Moessinger C. Jakobsson L. (2017). Neuropilin 1 binds PDGF-D and is a co-receptor in PDGF-D-PDGFRβ signaling. *J. Cell Sci.* 130 1365–1378. 10.1242/jcs.200493 28254885

[B133] Musumeci F. Radi M. Brullo C. Schenone S. (2012). Vascular endothelial growth factor (VEGF) receptors: drugs and new inhibitors. *J. Med. Chem.* 55 10797–10822. 10.1021/jm301085w 23098265

[B134] Nag S. Manias J. L. Stewart D. J. (2009). Expression of endothelial phosphorylated caveolin-1 is increased in brain injury. *Neuropathol. Appl. Neurobiol.* 35 417–426. 10.1111/j.1365-2990.2008.01009.x 19508446

[B135] Nakayama A. Nakayama M. Turner C. J. Höing S. Lepore J. J. Adams R. H. (2013). Ephrin-B2 controls PDGFRβ internalization and signaling. *Genes Dev.* 27 2576–2589. 10.1101/gad.224089.113 24298057 PMC3861671

[B136] Nguyen L. N. Ma D. Shui G. Wong P. Cazenave-Gassiot A. Zhang X. (2014). Mfsd2a is a transporter for the essential omega-3 fatty acid docosahexaenoic acid. *Nature* 509 503–506. 10.1038/nature13241 24828044

[B137] Nichol D. Shawber C. Fitch M. J. Bambino K. Sharma A. Kitajewski J. (2010). Impaired angiogenesis and altered Notch signaling in mice overexpressing endothelial Egfl7. *Blood* 116 6133–6143. 10.1182/blood-2010-03-274860 20947685 PMC3031397

[B138] Oladipupo S. S. Kabir A. U. Smith C. Choi K. Ornitz D. M. (2018). Impaired tumor growth and angiogenesis in mice heterozygous for Vegfr2 (Flk1). *Sci. Rep.* 8 1–10. 10.1038/s41598-018-33037-2 30283071 PMC6170482

[B139] Orellana J. A. Figueroa X. F. Sánchez H. A. Contreras-Duarte S. Velarde V. Sáez J. C. (2011). Hemichannels in the neurovascular unit and white matter under normal and inflamed conditions. *CNS Neurol. Disord. Drug Targets* 10 404–414. 10.2174/187152711794653869 21288190

[B140] Oshikawa J. Urao N. Kim H. W. Kaplan N. Razvi M. McKinney R. (2010). Extracellular SOD-derived H2O2 promotes VEGF signaling in caveolae/lipid rafts and post-ischemic angiogenesis in mice. *PLoS One* 5:e0010189. 10.1371/journal.pone.0010189 20422004 PMC2858087

[B141] Paik J. Skoura A. Chae S. Cowan A. E. Han D. K. Proia R. L. (2004). Sphingosine 1-phosphate receptor regulation of N-cadherin mediates vascular stabilization. *Genes Dev.* 114 2392–2403. 10.1101/gad.1227804 15371328 PMC522989

[B142] Pani B. Singh B. B. (2009). Lipid rafts/caveolae as microdomains of calcium signaling. *Cell Calcium* 45 625–633. 10.1016/j.ceca.2009.02.009 19324409 PMC2695836

[B143] Paniagua-Herranz L. Gil-Redondo J. C. Queipo M. J. González-Ramos S. Boscá L. Pérez-Sen R. (2017). Prostaglandin E2 Impairs P2Y2/P2Y4 receptor signaling in cerebellar astrocytes via EP3 receptors. *Front. Pharmacol.* 8:937. 10.3389/fphar.2017.00937 29311938 PMC5743739

[B144] Paonessa F. Evans L. D. Solanki R. Larrieu D. Wray S. Hardy J. (2019). Microtubules deform the nuclear membrane and disrupt nucleocytoplasmic transport in Tau-mediated frontotemporal dementia. *Cell Rep.* 26 582.e–593.e. 10.1016/j.celrep.2018.12.085 30650353 PMC6335264

[B145] Patiño P. Parada E. Farré-Alins V. Molz S. Cacabelos R. Marco-Contelles J. (2016). Melatonin protects against oxygen and glucose deprivation by decreasing extracellular glutamate and Nox-derived ROS in rat hippocampal slices. *Neuro Toxicol.* 57 61–68. 10.1016/j.neuro.2016.09.002 27620136

[B146] Peppiatt C. M. Howarth C. Mobbs P. Attwell D. (2006). Bidirectional control of CNS capillary diameter by pericytes. *Nature* 443 1–5. 10.1038/nature05193 17036005 PMC1761848

[B147] Piazzini V. Landucci E. Graverini G. Pellegrini-Giampietro D. E. Bilia A. R. Bergonzi M. C. (2018). Stealth and cationic nanoliposomes as drug delivery systems to increase andrographolide BBB permeability. *Pharmaceutics* 10 1–19. 10.3390/pharmaceutics10030128 30104484 PMC6161272

[B148] Podar K. Anderson K. C. (2008). The pathophysiologic role of VEGF in hematologic malignancies?: therapeutic implications review in translational hematology the pathophysiologic role of VEGF in hematologic malignancies?: therapeutic implications. *Clin. Res.* 105 1383–1395. 10.1182/blood-2004-07-2909 15471951

[B149] Pulgar V. M. (2019). Transcytosis to cross the blood brain barrier, new advancements and challenges. *Front. Neurosci.* 13:1019. 10.3389/fnins.2018.01019 30686985 PMC6337067

[B150] Qian Z. M. Li H. Sun H. Ho K. (2002). Targeted drug delivery via the transferrin receptor-mediated endocytosis pathway. *Pharmacol. Rev.* 54 561–587. 10.1124/pr.54.4.561 12429868

[B151] Quancard J. Bollbuck B. Janser P. Angst D. Berst F. Buehlmayer P. (2012). A potent and selective S1P1 antagonist with efficacy in experimental autoimmune encephalomyelitis. *Chem. Biol.* 19 1142–1151. 10.1016/j.chembiol.2012.07.016 22999882

[B152] Rausch V. Bostrom J. R. Park J. Bravo I. R. Feng Y. Hay D. C. (2019). The hippo pathway regulates caveolae expression and mediates flow response via caveolae. *Curr. Biol.* 29:242-255.e6. 10.1016/j.cub.2018.11.066 30595521 PMC6345631

[B153] Razani B. Engelman J. A. Wang X. B. Schubert W. Zhang X. L. Marks C. B. (2001). Caveolin-1 null mice are viable but show evidence of hyperproliferative and vascular abnormalities. *J. Biol. Chem.* 276 38121–38138. 10.1074/jbc.M105408200 11457855

[B154] Reeves P. M. Kang Y. L. Kirchhausen T. (2016). Endocytosis of ligand-activated Sphingosine 1-Phosphate receptor 1 mediated by the Clathrin-pathway. *Traffic* 17 40–52. 10.1111/tra.12343 26481905 PMC4688054

[B155] Reinitz A. DeStefano J. Ye M. Wong A. D. Searson P. C. (2015). Human brain microvascular endothelial cells resist elongation due to shear stress. *Microvasc. Res.* 99 8–18. 10.1016/j.mvr.2015.02.008 25725258 PMC4426013

[B156] Reyahi A. Nik A. M. Ghiami M. Gritli-Linde A. Pontén F. Johansson B. R. (2015). Foxf2 is required for brain pericyte differentiation and development and maintenance of the blood-brain barrier. *Dev. Cell* 34 19–32. 10.1016/j.devcel.2015.05.008 26120030

[B157] Riganti L. Antonucci F. Gabrielli M. Prada I. Giussani P. Viani P. (2016). Sphingosine-1-Phosphate (S1P) impacts presynaptic functions by regulating Synapsin I localization in the presynaptic compartment. *J. Neurosci.* 36 4624–4634. 10.1523/JNEUROSCI.3588-15.2016 27098703 PMC6601834

[B158] Rochfort K. D. Collins L. E. Murphy R. P. Cummins P. M. (2014). Downregulation of blood-brain barrier phenotype by proinflammatory cytokines involves NADPH oxidase-dependent ROS generation: consequences for interendothelial adherens and tight junctions. *PLoS One* 9:e0101815. 10.1371/journal.pone.0101815 24992685 PMC4081725

[B159] Römer W. Pontani L. L. Sorre B. Rentero C. Berland L. Chambon V. (2010). Actin dynamics drive membrane reorganization and Scission in Clathrin-independent endocytosis. *Cell* 140 540–553. 10.1016/j.cell.2010.01.010 20178746

[B160] Ruiz M. Okada H. Dahlbäck B. (2017). HDL-associated ApoM is anti-apoptotic by delivering sphingosine 1-phosphate to S1P1 & S1P3 receptors on vascular endothelium. *Lipids Health Dis.* 16:36. 10.1186/s12944-017-0429-2 28179022 PMC5299634

[B161] Sabayan B. Buchem M. A. Van Sigurdsson S. Zhang Q. Harris T. B. Gudnason V. (2015). Cardiac hemodynamics are linked with structural and functional. *J. Am. Heart Assoc.* 4:e001294. 10.1161/JAHA.114.001294 25628405 PMC4330056

[B162] Sade H. Baumgartner C. Hugenmatter A. Moessner E. Freskga P. Niewoehner J. (2014). A human blood-brain barrier transcytosis assay reveals antibody transcytosis influenced by ph-dependent receptor binding. *PLoS One* 9:e0096340. 10.1371/journal.pone.0096340 24788759 PMC4005765

[B163] Sadeghian H. Lacoste B. Qin T. Toussay X. Rosa R. Oka F. (2018). Spreading depolarizations trigger caveolin-1–dependent endothelial transcytosis. *Ann. Neurol.* 84 409–423. 10.1002/ana.25298 30014540 PMC6153037

[B164] Sanchez T. Skoura A. Wu M. T. Casserly B. Harrington E. O. Hla T. (2007). Induction of vascular permeability by the sphingosine-1-phosphate receptor-2 (S1P2R) and its downstream effectors ROCK and PTEN. *Arterioscler. Thromb. Vasc. Biol.* 27 1312–1318. 10.1161/ATVBAHA.107.143735 17431187

[B165] Sanders Y. Y. Cui Z. Le Saux C. J. Horowitz J. C. Rangarajan S. Kurundkar A. (2015). SMAD-independent down-regulation of caveolin-1 by TGF-β: Effects on proliferation and survival of myofibroblasts. *PLoS One* 10:e0116995. 10.1371/journal.pone.0116995 25658089 PMC4319960

[B166] Sawamiphak S. Seidel S. Essmann C. L. Wilkinson G. A. Pitulescu M. E. Acker T. (2010). Ephrin-B2 regulates VEGFR2 function in developmental and tumour angiogenesis. *Nature* 465 487–491. 10.1038/nature08995 20445540

[B167] Schreibelt G. Kooij G. Reijerkerk A. van Doorn R. Gringhuis S. I. van der Pol S. (2007). Reactive oxygen species alter brain endothelial tight junction dynamics via RhoA. PI3 kinase, and PKB signaling. *FASEB J.* 21 3666–3676. 10.1096/fj.07-8329com 17586731

[B168] Shalaby F. Janet R. Yamaguchi T. P. Gertsenstein M. Wu X. F. Breitman M. L. (1995). Failure of blood-island formation and vasculogenesis in Flk-1-deficient mice. *Nature* 376 62–66. 10.1038/376062a0 7596435

[B169] Shang D. Peng T. Gou S. Li Y. Wu H. Wang C. (2016). High mobility group box protein 1 boosts endothelial albumin transcytosis through the RAGE/Src/Caveolin-1 pathway. *Sci. Rep.* 6 1–12. 10.1038/srep32180 27572515 PMC5004123

[B170] Sharghi-Namini S. Tan E. Ong L. L. S. Ge R. Asada H. H. (2014). Dll4-containing exosomes induce capillary sprout retraction in a 3D microenvironment. *Sci. Rep.* 4 1–8. 10.1038/srep04031 24504253 PMC3916896

[B171] Sheldon H. Sainson R. C. A. Sargent I. Li J.-L. Harris A. L. Heikamp E. (2010). New mechanism for Notch signaling to endothelium at a distance by Delta-like 4 incorporation into exosomes. *Blood* 116 2385–2394. 10.1182/blood-2009-08-239228 20558614

[B172] Shen J. Xu G. Zhu R. Yuan J. Ishii Y. Hamashima T. (2018). PDGFR-β restores blood-brain barrier functions in a mouse model of focal cerebral ischemia. *J. Cereb. Blood Flow Metab.* 39 1501–1515. 10.1177/0271678X18769515 29629621 PMC6681529

[B173] Shepherd J. Fisher M. Welford A. McDonald D. M. Kanthou C. Tozer G. M. (2017). The protective role of sphingosine-1-phosphate against the action of the vascular disrupting agent combretastatin A-4 3-O-phosphate. *Oncotarget* 8 95648–95661. 10.18632/oncotarget.21172 29221156 PMC5707050

[B174] Shimato S. Anderson L. M. Asslaber M. Bruce J. N. Canoll P. Anderson D. E. (2013). Inhibition of Caveolin-1 Restores Myeloid Cell Function in Human Glioblastoma. *PLoS One* 8:e0077397. 10.1371/journal.pone.0077397 24130882 PMC3793958

[B175] Shutter J. R. Scully S. Fan W. Richards W. G. Kitajewski J. Deblandre G. A. (2000). D114, a novel Notch ligand expressed in arterial endothelium. *Genes Dev.* 14 1313–1318. 10.1101/gad.14.11.1313 10837024 PMC316657

[B176] Song J. Dagan A. Yakhtin Z. Gatt S. Riley S. Rosen H. (2017). The novel sphingosine-1-phosphate receptors antagonist AD2900 affects lymphocyte activation and inhibits T-cell entry into the lymph nodes. *Oncotarget* 8 53563–53580. 10.18632/oncotarget.18626 28881832 PMC5581131

[B177] Song L. Ge S. Pachter J. S. (2007). Caveolin-1 regulates expression of junction-associated proteins in brain microvascular endothelial cells. *Blood* 109 1515–1523. 10.1182/blood-2006-07-034009 17023578 PMC1794065

[B178] Stebbins M. J. Wilson H. K. Canfield S. G. Qian T. Palecek S. P. Shusta E. V. (2015). Differentiation and characterization of human pluripotent stem cell-derived brain microvascular endothelial cells. *Methods* 101 93–102. 10.1016/j.ymeth.2015.10.016 26518252 PMC4848177

[B179] Streets A. M. Huang Y. (2013). Chip in a lab: Microfluidics for next generation life science research. *Biomicrofluidics* 7 1–23. 10.1063/1.4789751 23460772 PMC3574129

[B180] Tanaka S. Suto A. Ikeda K. Sanayama Y. Nakagomi D. Iwamoto T. (2013). Alteration of circulating miRNAs in SSc: MiR-30b regulates the expression of PDGF receptor β. *Rheumatology* 52 1963–1972. 10.1093/rheumatology/ket254 23893664

[B181] Teichert M. Milde L. Holm A. Stanicek L. Gengenbacher N. Savant S. (2017). Pericyte-expressed Tie2 controls angiogenesis and vessel maturation. *Nat. Commun.* 8 1–12. 10.1038/ncomms16106 28719590 PMC5520106

[B182] Thanabalasundaram G. Schneidewind J. Pieper C. Galla H. J. (2011). The impact of pericytes on the blood-brain barrier integrity depends critically on the pericyte differentiation stage. *Int. J. Biochem. Cell Biol.* 43 1284–1293. 10.1016/j.biocel.2011.05.002 21601005

[B183] Thornton C. Baburamani A. A. Kichev A. Hagberg H. (2017). Oxidative stress and endoplasmic reticulum (ER) stress in the development of neonatal hypoxic–ischaemic brain injury. *Biochem. Soc. Trans.* 45 1067–1076. 10.1042/bst20170017 28939695 PMC5652227

[B184] Tien T. Muto T. Barrette K. Challyandra L. Roy S. (2014). Downregulation of Connexin 43 promotes vascular cell loss and excess permeability associated with the development of vascular lesions in the diabetic retina. *Mol. Vis.* 20 732–741. 24940027 PMC4043608

[B185] Tillet E. Vittet D. Féraud O. Moore R. Kemler R. Huber P. (2005). N-cadherin deficiency impairs pericyte recruitment, and not endothelial differentiation or sprouting, in embryonic stem cell-derived angiogenesis. *Exp. Cell Res.* 310 392–400. 10.1016/j.yexcr.2005.08.021 16202998

[B186] Tiruppathi C. Naqvi T. Wu Y. Vogel S. M. Minshall R. D. Malik A. B. (2004). Albumin mediates the transcytosis of myeloperoxidase by means of caveolae in endothelial cells. *Proc. Natl. Acad. Sci. U.S.A.* 101 7699–7704. 10.1073/pnas.0401712101 15136724 PMC419669

[B187] Tiwary S. Morales J. E. Kwiatkowski S. C. Lang F. F. Rao G. Mccarty J. H. (2018). Metastatic brain tumors disrupt the blood-brain barrier and alter lipid metabolism by inhibiting expression of the endothelial cell fatty acid transporter Mfsd2a. *Sci. Rep.* 8:8267. 10.1038/s41598-018-26636-6 29844613 PMC5974340

[B188] Tominaga N. Kosaka N. Ono M. Katsuda T. Yoshioka Y. Tamura K. (2015). Brain metastatic cancer cells release microRNA-181c-containing extracellular vesicles capable of destructing blood-brain barrier. *Nat. Commun.* 6:7716. 10.1038/ncomms7716 25828099 PMC4396394

[B189] Toyama K. Spin J. M. Deng A. C. Huang T. T. Wei K. Wagenhäuser M. U. (2018). MicroRNA-mediated therapy modulating blood-brain barrier disruption improves vascular cognitive impairment. *Arterioscler. Thromb. Vasc. Biol.* 38 1392–1406. 10.1161/ATVBAHA.118.310822 29650692

[B190] Underly R. G. Levy M. Hartmann D. A. Grant R. I. Watson A. N. Shih A. Y. (2017). Pericytes as inducers of rapid, matrix metalloproteinase-9-dependent capillary damage during ischemia. *J. Neurosci.* 37 129–140. 10.1523/JNEUROSCI.2891-16.2016 28053036 PMC5214626

[B191] Valable S. Montaner J. Bellail A. Berezowski V. Brillault J. Cecchelli R. (2005). VEGF-induced BBB permeability is associated with an MMP-9 activity increase in cerebral ischemia: Both effects decreased by Ang-1. *J. Cereb. Blood Flow Metab.* 25 1491–1504. 10.1038/sj.jcbfm.9600148 15902195

[B192] Van der Goes A. Wouters D. Susanne M. Van der Pol S. Huizinga R. Ronken E. (2002). Reactive oxygen species enhance the migration of monocytes across the blood-brain barrier in vitro. *FASEB J.* 15 1852–1854. 10.1096/fj.00-0881fje 11481252

[B193] Vézina A. Charfi C. Zgheib A. Annabi B. (2018). Cerebrovascular Angiogenic reprogramming upon LRP1 repression: impact on Sphingosine-1-Phosphate-mediated signaling in brain endothelial cell chemotactism. *Mol. Neurobiol.* 55 3551–3563. 10.1007/s12035-017-0614-3 28516428

[B194] Villaseñor R. Lampe J. Schwaninger M. Collin L. (2019). Intracellular transport and regulation of transcytosis across the blood–brain barrier. *Cell. Mol. Life Sci.* 76 1081–1092. 10.1007/s00018-018-2982-x 30523362 PMC6513804

[B195] Wang F. Yamauchi M. Muramatsu M. Osawa T. Tsuchida R. Shibuya M. (2011). RACK1 regulates VEGF/Flt1-mediated cell migration via activation of a PI3K/Akt pathway. *J. Biol. Chem.* 286 9097–9106. 10.1074/jbc.M110.165605 21212275 PMC3058986

[B196] Wang H. U. Chen Z. F. Anderson D. J. (1998). Molecular distinction and angiogenic interaction between embryonic arteries and veins revealed by ephrin-B2 and its receptor Eph-B4. *Cell* 93 741–753. 10.1016/S0092-8674(00)81436-1 9630219

[B197] Werion A. Joris V. Hepp M. Papasokrati L. Marique L. De Ville De Goyet C. (2016). Pioglitazone, a PPARγ agonist, upregulates the expression of Caveolin-1 and Catalase, essential for thyroid cell homeostasis: a clue to the pathogenesis of Hashimoto’s Thyroiditis. *Thyroid* 26 1320–1331. 10.1089/thy.2015.0625 27324467

[B198] Williams C. K. Li J. L. Murga M. Harris A. L. Tosato G. (2006). Up-regulation of the Notch ligand Delta-like 4 inhibits VEGF-induced endothelial cell function. *Blood* 107 931–939. 10.1182/blood-2005-03-1000 16219802 PMC1895896

[B199] Williams J. J. L. Alotaiq N. Mullen W. Burchmore R. Liu L. Baillie G. S. (2018). Interaction of suppressor of cytokine signalling 3 with cavin-1 links SOCS3 function and cavin-1 stability. *Nat. Commun.* 9:168. 10.1038/s41467-017-02585-y 29330478 PMC5766592

[B200] Winkler E. A. Bell R. D. Zlokovic B. V. (2010). Pericyte-specific expression of PDGF beta receptor in mouse models with normal and deficient PDGF beta receptor signaling. *Mol. Neurodegen.* 5 1–11. 10.1186/1750-1326-5-32 20738866 PMC2936891

[B201] Witmer A. N. Blijswijk B. C. Van Noorden C. J. F. Van Vrensen G. F. J. M. (2004). In vivo angiogenic phenotype of endothelial cells and pericytes induced by vascular endothelial growth factor-A. *J. Histochem. Cytochem.* 52 39–52. 10.1177/002215540405200105 14688216

[B202] Wu C. Wu Y. Jin Y. Zhu P. Shi W. Li J. (2019). Endosomal/lysosomal location of organically modified silica nanoparticles following caveolae-mediated endocytosis. *RSC Adv.* 9 13855–13862. 10.1039/c9ra00404a PMC9063904 35519602

[B203] Xi T. Jin F. Zhu Y. Wang J. Tang L. Wang Y. (2017). MicroRNA-126-3p attenuates blood-brain barrier disruption, cerebral edema and neuronal injury following intracerebral hemorrhage by regulating PIK3R2 and Akt. *Biochem. Biophys. Res. Commun.* 494 144–151. 10.1016/j.bbrc.2017.10.064 29042193

[B204] Xie H. Lu W. C. (2018). Inhibition of transient receptor potential vanilloid 4 decreases the expressions of caveolin-1 and caveolin-2 after focal cerebral ischemia and reperfusion in rats. *Neuropathology* 38 337–346. 10.1111/neup.12469 29665111

[B205] Yakoub A. M. Sadek M. (2018). Development and characterization of human cerebral organoids: an optimized protocol. *Cell Transplant.* 27 393–406. 10.1177/0963689717752946 29749250 PMC6038047

[B206] Yanagida K. Liu C. H. Faraco G. Galvani S. Smith H. K. Burg N. (2017). Size-selective opening of the blood-brain barrier by targeting endothelial sphingosine 1-phosphate receptor 1. *Proc. Natl. Acad. Sci. U.S.A.* 114 4531–4536. 10.1073/pnas.1618659114 28396408 PMC5410849

[B207] Yao Y. Chen Z. L. Norris E. H. Strickland S. (2014). Astrocytic laminin regulates pericyte differentiation and maintains blood brain barrier integrity. *Nat. Commun.* 5:4413. 10.1038/ncomms4413 24583950 PMC3992931

[B208] Ye M. Sanchez H. M. Hultz M. Yang Z. Bogorad M. Wong A. D. (2014). Brain microvascular endothelial cells resist elongation due to curvature and shear stress. *Sci. Rep.* 4 1–6. 10.1038/srep04681 24732421 PMC3986701

[B209] Zeng Y. Liu X. Tarbell J. Fu B. (2015). Sphingosine 1-phosphate induced synthesis of glycocalyx on endothelial cells. *Exp. Cell Res.* 339 90–95. 10.1016/j.yexcr.2015.08.013 26364737

[B210] Zhang M. Lee S. J. An C. Xu J. F. Joshi B. Nabi I. R. (2011). Caveolin-1 mediates Fas-BID signaling in hyperoxia-induced apoptosis. *Free Radical Biol. Med.* 50 1252–1262. 10.1016/j.freeradbiomed.2011.02.031 21382479 PMC4134776

[B211] Zhang X. Kim K. M. (2017). Multifactorial regulation of g protein-coupled receptor endocytosis. *Biomol. Ther.* 25 26–43. 10.4062/biomolther.2016.186 28035080 PMC5207461

[B212] Zhao Z. Zlokovic B. V. (2014). Blood-Brain Barrier: A Dual Life of MFSD2A? *Neuron* 82 728–730. 10.1016/j.neuron.2014.05.012 24853933 PMC4114515

[B213] Zheng B. Yin W. N. Suzuki T. Zhang X. H. Zhang Y. Song L. L. (2017). Exosome-Mediated miR-155 Transfer from Smooth Muscle Cells to Endothelial Cells Induces Endothelial Injury and Promotes Atherosclerosis. *Mol. Ther.* 25 1279–1294. 10.1016/j.ymthe.2017.03.031 28408180 PMC5475247

[B214] Zhou H. J. Qin L. Zhang H. Tang W. Ji W. He Y. (2016). Endothelial exocytosis of angiopoietin-2 resulting from CCM3 deficiency contributes to cerebral cavernous malformation. *Nat. Med.* 22 1033–1042. 10.1038/nm.4169 27548575 PMC5014607

[B215] Zhou Q. Zheng X. Chen L. Xu B. Yang X. Jiang J. (2016). Smad2/3/4 pathway contributes to TGF-β-induced MiRNA-181b expression to promote gastric cancer metastasis by targeting Timp3. *Cell. Physiol. Biochem.* 39 453–466. 10.1159/000445638 27383203

[B216] Zhou T. Zheng Y. Sun L. Badea S. R. Jin Y. Liu Y. (2019). Microvascular endothelial cells engulf myelin debris and promote macrophage recruitment and fibrosis after neural injury. *Nat. Neurosci* 22 421–435. 10.1038/s41593-018-0324-9 30664769 PMC6913093

[B217] Zhou X. Wu Q. Lu Y. Zhang X. Lv S. Shao J. (2019). Crosstalk between soluble PDGF-BB and PDGFRβ promotes astrocytic activation and synaptic recovery in the hippocampus after subarachnoid hemorrhage. *FASEB J.* 33 9588–9601. 10.1096/fj.201900195r 31162947

